# Design of mechanical-robust phosphorescence materials through covalent click reaction

**DOI:** 10.1038/s41467-023-40451-2

**Published:** 2023-08-05

**Authors:** Rui Tian, Shuo Gao, Kaitao Li, Chao Lu

**Affiliations:** 1 State Key Laboratory of Chemical Resource Engineering, Beijing University of Chemical Technology, North, Third Ring Road 15, Chaoyang District, Beijing, China; 2 Green Catalysis Center, College of Chemistry, Zhengzhou University, No.100 Science Avenue, Zhengzhou, China

**Keywords:** Polymers, Polymers, Organic-inorganic nanostructures

## Abstract

It remains a great challenge to engineer materials with strong and stable interactions for the simultaneously mechanical-robust and room temperature phosphorescence-efficient materials. In this work, we demonstrate a covalent cross-linking strategy to engineer mechanical-robust room temperature phosphorescence materials through the B–O click reaction between chromophores, polyvinyl alcohol matrix and inorganic layered double hydroxide nanosheets. Through the covalent cross-linkage between the organic polyvinyl alcohol and inorganic layered double hydroxide, a polymeric composite with ultralong lifetime up to 1.45 s is acquired based on the inhibited non-radiative transition of chromophores. Simultaneously, decent mechanical strength of 97.9 MPa can be realized for the composite materials due to the dissipated loading stress through the covalent-bond-accommodated interfacial interaction. These cross-linked composites also exhibit flexibility, processability, scalability and phosphorescence responses towards the mechanical deformation. It is anticipated that the proposed covalent click reaction could provide a platform for the design and modulation of composites with multi-functionality and long-term durability.

## Abstract (web summary)

Materials with room temperature phosphorescence that are mechanically robust are desirable, but can be challenging to obtain. Here, the authors report the combination of poly(vinyl alcohol) and chromophore molecules with layered double hydroxides for room temperature phosphorescent materials.

## Introduction

Accompanied by the prosperous development of polymer-based room temperature phosphorescence (RTP) materials in the last decade, the research focus has been switched from the preparation of efficient RTP materials to the practical application development^1–6^. The current applications of polymer-based RTP materials impose great challenges of requirements for their strong mechanical properties, such as flexible sensors or integrated devices^7–9^. It is recommended that the polymer-based RTP materials could be stretchable, flexible, and robust upon mechanical deformation^10–12^. Noteworthy that the RTP performances of these polymer-based materials should not be compromised with the promotion of their mechanical properties. Therefore, it is essential to pursue feature materials with a perfect combination of decent RTP and mechanical properties.

Incorporating inorganic components into polymer-based RTP materials is highly appealing to meet the practical requirements. The inorganic components could provide a rigid environment for the amorphous polymer-based RTP materials, contributing to the restricted relaxation motions of chromophores and the reinforced mechanical properties of the polymer matrix^13–15^. Tian and Walther have pioneered the construction of inorganic-organic composites based on a bioinspired nacre-mimetic structure, exhibiting improved RTP and mechanical properties^16^. These efforts have drawn wide interests and concerns on the interactions between the organic and inorganic components in the composites. To meet these requirements, hydrogen bonds and electrostatic interaction have been generally introduced in the polymeric composites to enhance the interfacial interactions between multi components^17–19^. Disappointedly, hydrogen bonds and electrostatic interaction are generally hygroscopic. They could be hydrated by the invaded water, and thus RTP of the composites would be quenched or the relevant mechanical properties would be decreased^20–22^. Therefore, a reliable strategy to engineer materials with strong and stable interactions for the mechanical-robust RTP materials remains a contemporary challenge.

Covalent bonds with high energy and stability would offer an effective approach to solve the vulnerable interactions between inorganic and organic phases in the polymeric composites^23–25^. Covalent bonds could serve not only as the cross-linkages for rigidification, but also tolerate and dissipate the stress from the loading strain^26,27^. There is no good reason to disregard the covalent bonds in constructing mechanical-robust RTP materials. However, covalent reactions usually require sophisticated synthesis or modification of components to meet the specific requirements for covalent bonding. For example, Saito and coworkers have attached the boronic ester groups to the chain structure of a thermoplastic elastomer through a one-day reaction in the atmosphere of argon, in order to achieve the covalent cross-linking between the commodity inorganic fillers and polymer matrix^28^. These reaction requirements have interfered with the development of covalent bonding in constructing composite materials^29^. Encouragingly, click reaction has been known due to the excellent chemoselectivity, operational simplicity, high yields, and avoidance of by-products or harsh solvents^30,31^. Engineered click reactions could link the functional moieties to the desired location in a mild and modularized approach^32^. Click chemistry can effectively avoid complex synthesis procedures during covalent cross-linking, providing possibilities for the preparation of composite materials with covalent bonds.

**Fig. 1:**
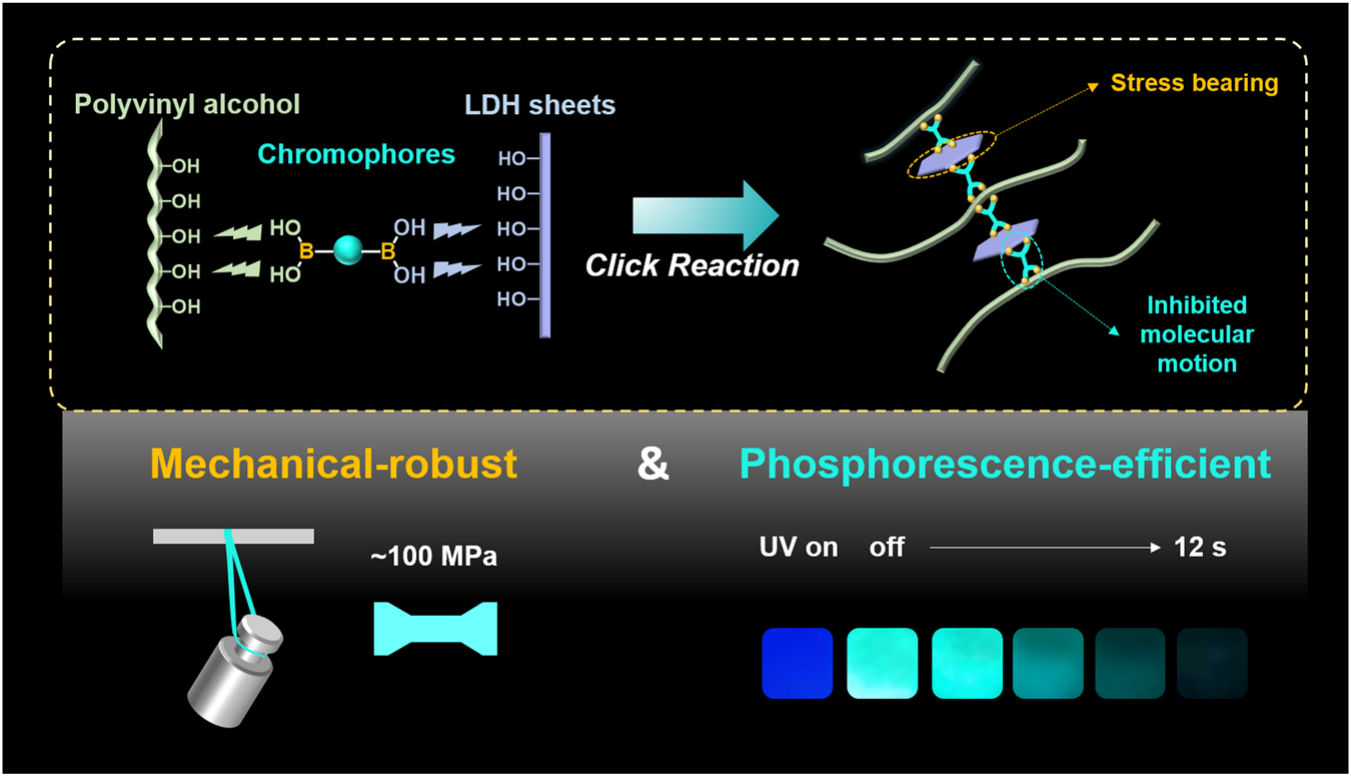
Schematic representation for the mechanical-robust room temperature phosphorescence materials. The enhanced RTP and mechanical properties originate from multiple covalent cross-linking through click reaction.

In this work, we have elaborately designed a click reaction between the chromophores with boronic acid groups, polyvinyl alcohol (PVA) matrix, and inorganic layered double hydroxide (LDHs) nanosheets with abundant hydroxyl groups to achieve a mechanical-robust RTP material (Fig. 1). A cross-linked network has been established based on the multiple covalent B‒O bonds. Such a rigid network has effectively restricted the molecular motions and non-radiative transition of chromophores, contributing to the boosted RTP performances with an ultralong lifetime to 1.45 s. Meanwhile, the as-prepared films show excellent mechanical robustness with a tensile strength up to 97.9 MPa, due to the accommodated interfacial interaction between the cross-linked inorganic and organic phases. The as-prepared composite films also exhibit decent scalability, flexibility, stretchability, and sensitive phosphorescence responses toward mechanical deformation. The perfect blend of the notably enhanced RTP and mechanical properties of the composite films demonstrate the power of covalent cross-linking between the chromophores, polymer matrix, and inorganic reinforcer. It is anticipated that the established covalent click reaction could provide possibilities for the design of practical optical devices with durability and application flexibility.

## Results

### Construction of the cross-linked organic-inorganic composite films

**Fig. 2:**
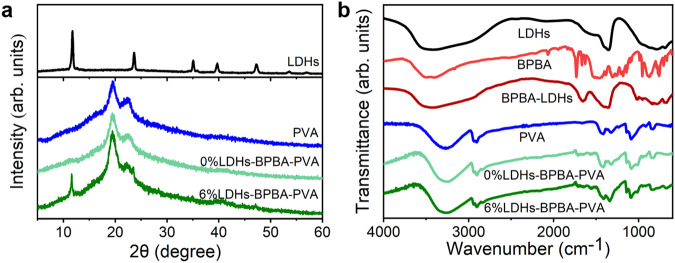
Structural studies for *x*% LDHs-BPBA-PVA composite films. **a** XRD patterns and **b** FT-IR spectra for LDHs, PVA, and *x*% LDHs-BPBA-PVA composite films.

To construct a covalent cross-linking network for mechanical-robust RTP material, active components with boronic acid or hydroxyl groups were selected elaborately. The 4,4’-biphenyldiboronic acid (BPBA) molecules with two boronic acid groups at the ends of biphenyl were used as chromophores (Supplementary Fig. 1a). PVA was selected as a polymer matrix due to its sufficient and tunable hydroxyl groups along with flexibility, easy-preparation and low-cost (Supplementary Fig. 1b)^33–35^. LDHs are a kind of inorganic layered materials with the divalent/trivalent metal species as metal-oxygen octahedral structure in the host layer, and abundant hydroxyl groups were attached to these octahedrons in the host layers (Supplementary Fig. 1c)^36^. MgAl-LDHs, with a ratio of Mg/Al as 1.77 and lateral size ~2 μm (Supplementary Fig. 2), were employed in this work to induce the B‒O click reaction with PVA and BPBA molecules. X-ray diffraction patterns (XRD) showed LDHs exhibited characteristic peaks at 11.9°, 23.5° and 34.8°, corresponding to (003), (006), (009) crystal planes of LDHs intercalated with CO_3_^2-^ (*d* = 0.74 nm), respectively (Fig. 2a, black line)^37,38^. The typical peak at 19.6° of PVA was assigned to the (101) semi-crystalline plane (Fig. 2a, blue line)^39^. Cross-linked *x*% LDHs-BPBA-PVA composite films were prepared through a mild and rapid click reaction under room temperature. The quantities of BPBA were about 0.1 wt%, and the contents of LDHs varied from 1 wt% to 15 wt%. The featured peaks of LDHs at 11.9° can be observed in the composite films, indicating the successful introduction of LDHs into the composite films. No shift could be observed for this characteristic peak of LDHs, suggesting that the BPBA molecules were attached on the surface of LDHs to bridge LDHs and PVA. It could be observed that the relative intensities of the typical peak at 19.6° of PVA were promoted gradually with the increased contents of LDHs in the *x*% LDHs-BPBA-PVA composite films (Supplementary Fig. 3a). The possible reason was that the amorphous chains of PVA went through a covalent cross-linking reaction, resulting in the densely packed molecular arrangement and the reduced amorphous phase^40,41^. Moreover, the pH value of the BPBA was reduced from 10.86 to 10.54 after the addition of LDHs and PVA, revealing the successful transformation of the boronic acid to the boronic ester through the B–O reaction^42^.

Fourier transform infrared (FT-IR) measurements were further performed to verify the interaction between the different components in the composite films. The main characteristic peaks of LDHs were observed at 3100‒3600 cm^−1^ and 1350‒1380 cm^−1^ (Fig. 2b, black line), representing the abundant hydroxyl groups in the LDH layers and the nitrate anions between the interlayers of LDHs, respectively^43^. In comparison with the BPBA and LDHs, the absorption peak of BPBA-LDHs became wider and stronger in the range of 1310‒1430 cm^−1^ (Fig. 2b, dark red line), suggesting the formation of B‒O bond between LDHs and BPBA molecules. PVA showed the characteristic peak around 3260 cm^−1^, attributed to the O‒H stretching (Fig. 2b, blue line). This peak of PVA moved to the higher wavenumber after the reaction with LDHs and BPBA (Fig. 2b and Supplementary Fig. 3b), suggesting the reduced amounts of hydroxyl groups to form the cross-linked network^44^. Moreover, the absorption bands in the range of 1310 to 1430 cm^−1^ were intensified for the *x*% LDHs-BPBA-PVA composite films, which could be assigned to the formation of B‒O stretching vibration^45^. The new peak at 1030 cm^−1^, attributed to the B‒O‒C bond^46^, also verified the successful cross-linkage between BPBA, LDHs, and PVA in the composite films. These results demonstrated the success of the click reaction and the establishment of a rigid covalent network between BPBA, LDHs and PVA for the inorganic-organic *x*% LDHs-BPBA-PVA composite films. X-ray photoelectron spectroscopy (XPS) was implemented to study the variations of the coordination environment for B after the preparation of the composites. It could be observed from the B 1 *s* spectra that the binding energy shifted from 191.80 to 191.94 eV after the BPBA was localized in the composite films (Supplementary Fig. 4a and 4b). The B 1 *s* spectra could be further deconvoluted into the B−(OH)_2_ (192.00 eV) and B−C (191.70 eV) bonds in the BPBA molecules, while B−O−C (192.40 eV), B−C (191.50 eV) and B−O−M(Al) (189.50 eV) could be observed in the composites. These results demonstrated the varied coordination environment of B after the interaction with LDHs and PVA^47–49^. Moreover, the new peaks of O−C (533.20 eV) and O−Al (531.30 eV) in O 1 *s* spectra, and C−O (285.70 eV) in C 1 *s* spectra (Supplementary Fig. 4c−f), indicated that BPBA molecules were incorporated in the composite films^50,51^. In addition, a scanning electron microscope and energy dispersive spectrometer were employed for the composite films. The characteristic Mg and Al elements from LDHs were uniformly distributed throughout the 6% LDHs-BPBA-PVA films in the *x-y* plane and along *z* direction from top-view and side-view images (Supplementary Fig. 5), validating the incorporation of LDHs into the composite films.

### Boosted RTP behaviors of the *x*% LDHs-BPBA-PVA films

**Fig. 3:**
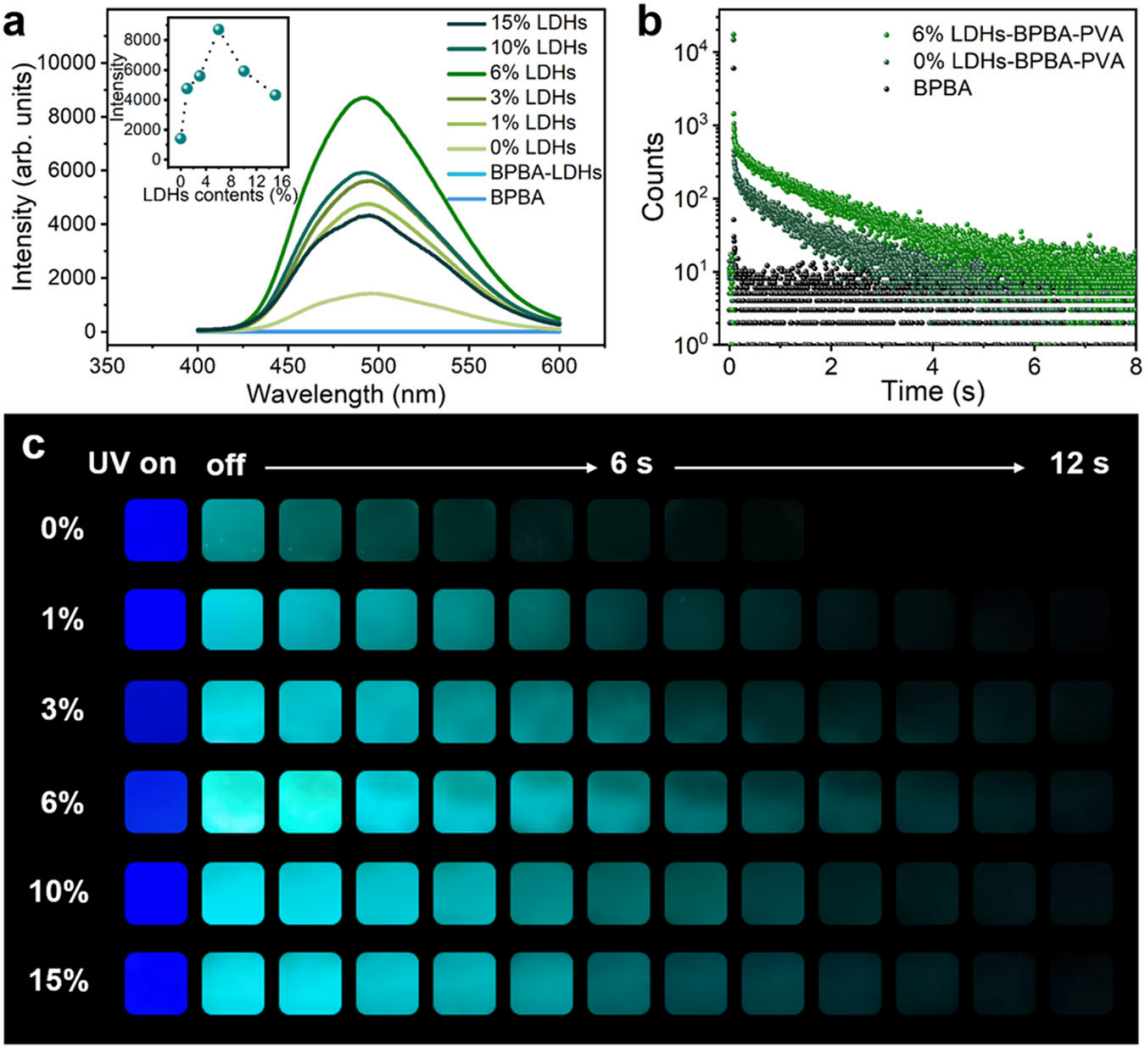
RTP behaviors of *x*% LDHs-BPBA-PVA films. **a** Phosphorescent emission spectra of *x*% LDHs-BPBA-PVA films, and the inset showed the variations of RTP intensities for *x*% LDHs-BPBA-PVA films. **b** Phosphorescence lifetimes of BPBA, BPBA-PVA, and 6% LDHs-BPBA-PVA films. **c** Photographs of *x*% LDHs-BPBA-PVA composite films under irradiation of 280-nm UV LED for 3 s and at different time intervals after the removal of UV irradiation (the concentration of BPBA were maintained at 0.1 wt% for all the samples, and the contents of LDHs varied from 0 wt % to 15 wt% of PVA).

The pure BPBA molecules showed rather weak blue fluorescence at 327 nm and green phosphorescence emissions at 495 nm (Fig. 3a). The BPBA molecules were covalently bonded to the non-emissive LDHs through the B‒O click reaction, and the LDHs-BPBA composites exhibited decent fluorescence and phosphorescence emissions (Supplementary Fig. 6). After the successful establishment of multiple covalent bonds between BPBA, LDHs and PVA, the *x*% LDHs-BPBA-PVA composite films showed the typical fluorescence and phosphorescence emissions as BPBA molecules. The fluorescence behaviors of the *x*% LDHs-BPBA-PVA composite films were significantly promoted (Supplementary Fig. 7). The fluorescence quantum yield for the 6% LDHs-BPBA-PVA film was 44.59%, which is much superior to that value of 29.11% for the BPBA-PVA film and 2.94% for the BPBA molecules (Supplementary Table 1). The fluorescence lifetime was also promoted from 1.29 ns for BPBA molecules to the 2.80 ns for the 6% LDHs-BPBA-PVA film (Supplementary Fig. 8). Impressively promoted RTP performances could be observed for the *x*% LDHs-BPBA-PVA composite films, in comparison with the BPBA, BPBA-LDHs, and BPBA-PVA film (Fig. 3a). The optimum 6% LDHs-BPBA-PVA film showed the highest RTP emissions, which was approximately fivefolds higher than that of BPBA-PVA film (*x*% = 0 wt%, Fig. 3a, inset). The 6% LDHs-BPBA-PVA film exhibited the longest lifetime of 1.45 s and a phosphorescence quantum yield of 3.92% (Fig. 3b and Supplementary Table 1). Note that such a boosted phosphorescence behavior was achieved with a doping content of BPBA for only 0.1 wt%, which is 10- to 20-folds lower than that in the previous reports^52,53^. More importantly, decent phosphorescence promotion could be acquired for the composites in the presence of LDHs, while the pristine phosphorescence lifetimes for the BPBA molecules and BPBA-PVA film were only 7 ms and 0.899 s, respectively. The fluorescence and RTP behaviors of the *x*% LDHs-BPBA-PVA films could be visualized under the excitation of UV light. The *x*% LDHs-BPBA-PVA films exhibited deep blue fluorescence under the UV light of 280 nm. Note that intense cyan phosphorescence emissions, with an afterglow time up to 12 s, could be traced by the naked eyes when the UV lamp was turned off (Fig. 3c). As a comparison, the afterglow of BPBA-PVA film without LDHs only lasted for 7 s, and no afterglow could be visualized for the BPBA molecules. These results demonstrated that the fluorescence and phosphorescence behaviors of BPBA could be promoted and reinforced by the rigidified cross-linking network with multiple covalent bonds between BPBA, PVA, and inorganic LDHs.

The origin of phosphorescence was ascribed to the BPBA molecules which featured with π-π* transition in the biphenyl moiety and n-π* transition from O atoms in the boronic acid groups to phenyl moiety^54^. To explain the mechanism for the boosted RTP behaviors for the cross-linked *x*% LDHs-BPBA-PVA films, a series of UV-Vis spectra were implemented for these composites. The absorbance of LDHs-BPBA-PVA at 350 nm was largely promoted than BPBA-PVA, while no absorption could be observed for the BPBA or the LDH, PVA components (Supplementary Fig. 9). The increased absorbance could be ascribed to the possible n→π* transition for the BPBA chromophores. These results verified the effect of the B‒O covalent network on promoting the intersystem crossing (ISC) process in the LDHs-BPBA-PVA composites. The dynamic photophysical parameters were calculated for validation. It could be observed that the rate constants for the non-radiative transition of phosphorescence was reduced from 1.07 s^−1^ for BPBA-PVA to 0.64 s^−1^ for 6% LDHs-BPBA-PVA, along with the promoted radiative transition rates and boosted phosphorescence emissions (Supplementary Table 2). According to these results, we reasonably believed that the effect of PVA matrix on inhibiting the molecular relaxation of BPBA was limited. Upon the introduction of inorganic LDHs, the multiple B‒O covalent bonds between PVA chains, BPBA molecules, and LDHs nanosheets effectively restricted molecular motion and inhibited the non-radiative decay of the excited triplet excitons for the phosphorescence emissions. Moreover, the rate constants for the ISC process of the composite films were improved from 1.03 × 10^7^ to 1.40 × 10^7^ s^−1^ after the introduction of LDHs. In addition, the excellent oxygen barrier performances of LDHs could also prevent the diffusion of ambient oxygen in the membrane and suppress the quenching of triplet excitons for the promoted RTP efficiency^55,56^. Therefore, we have efficiently promoted the RTP performances of organic chromophores and polymer-based composites through the introduction of LDHs and the corresponding rigid covalent cross-linkages.

### Regulation of the B‒O covalent bonds

**Fig. 4:**
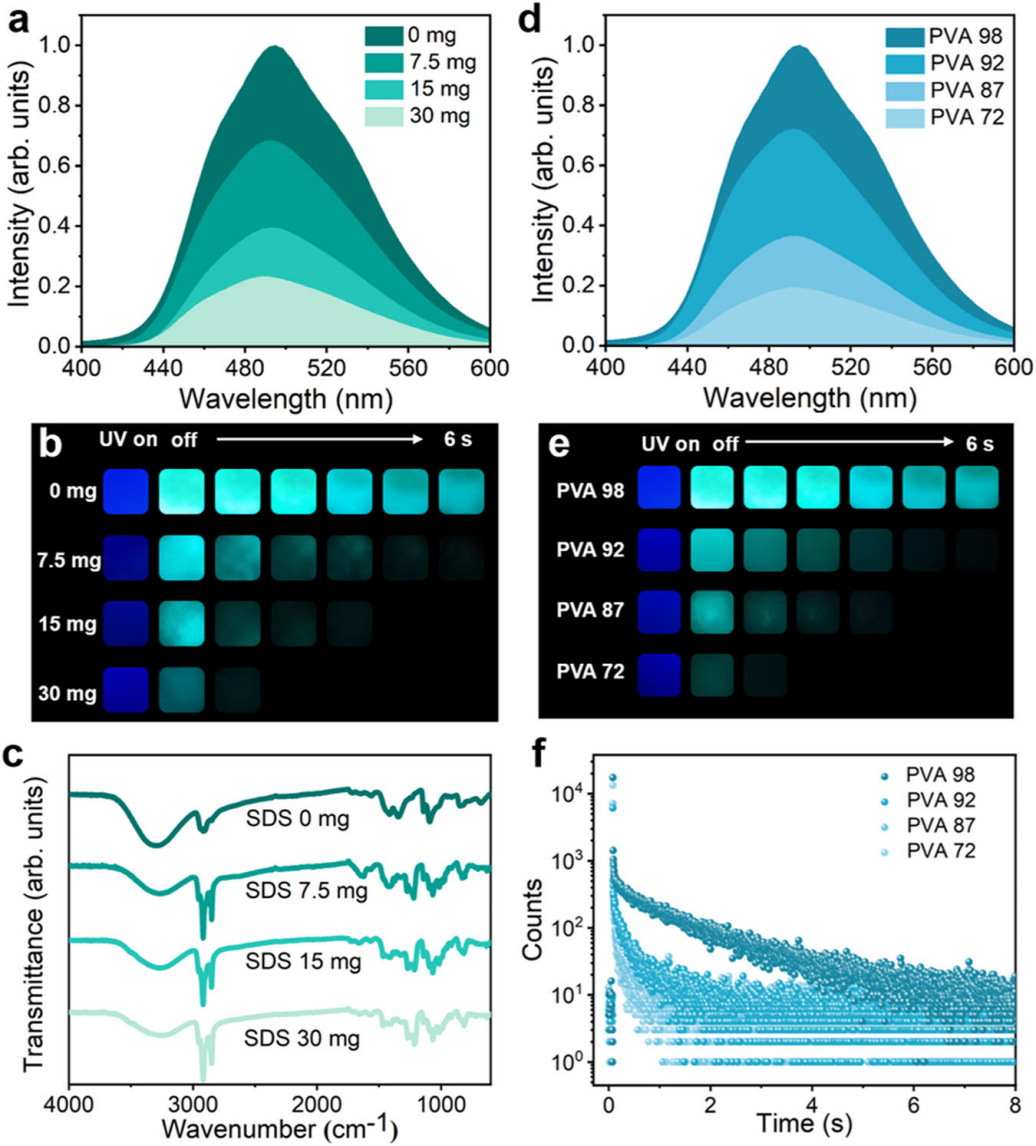
RTP behaviors of 6% LDHs-BPBA-PVA films with tunable B‒O covalent bonds. **a** Normalized phosphorescent emission spectra, **b** photographs of the afterglow, and **c** FT-IR spectra for 6% LDHs-BPBA-PVA films before and after the modification of LDHs by SDS. **d** Normalized phosphorescent emission spectra, **e** photographs of the afterglow and **f** RTP lifetimes for 6% LDHs-BPBA-PVA films with varied alcoholysis degree of PVA from 72%, 87%, 92% to 98% (the concentration of BPBA were maintained at 0.1 wt% for all the samples, and the contents of LDHs was 6 wt%).

To validate the function of B‒O covalent bonds in constructing the rigid network and facilitating the RTP performances, we have adjusted the quantities of hydroxyl groups in PVA and LDHs, respectively. First, sodium dodecyl sulfate (SDS) was used to modify the surface of LDHs. The active sites of hydroxyl groups in LDHs were partially occupied by the SDS, instead of the interaction with boronic acid groups from chromophores (Supplementary Fig. 10). The pristine zeta potential value of LDHs was measured as 40.93 mV, however, this value was decreased to 8.46 mV after the interaction with anionic surfactant SDS (Supplementary Fig. 11). Accordingly, the SDS attached on the surface of LDHs could occupy the active sites of hydroxyl groups, affecting the interaction between LDHs and chromophores with boronic acid groups. Both the phosphorescence and fluorescence intensities of 6% LDHs-BPBA-PVA composite films were distinctly weakened with the increased addition of SDS (Fig. 4a and Supplementary Fig. 12). When the weight ratio of SDS and LDHs reached 1:1 (weight of 30 mg), the phosphorescence intensity of the 6% LDHs-BPBA-PVA composite film decreased to only 23% of the pristine value, with the observable afterglow of 2 s after turning off the UV lamp (Fig. 4b). The reason to the decay of 6% LDHs-BPBA-PVA composite induced by the SDS was studied by the FT-IR and XRD measurements. The vibration peaks of B‒O stretching at 1310‒1430 cm^−1^ for LDHs-BPBA-PVA films decreased gradually with the increased addition of SDS (Fig. 4c). The occupied hydroxyl groups by SDS in LDHs also led to the decreased degree of cross-linkages in the LDHs-BPBA-PVA composite films, manifested as the lowered peak intensity at 19.6° in XRD patterns (Supplementary Fig. 13). These phenomena indicated that the addition of SDS resulted in the reduction of hydroxyl groups in LDHs and the decreased active sites for the click reaction to construct the LDHs-BPBA-PVA films, responsible for the lessened covalent bonds for the cross-linked network and the deterioration of RTP performance for the materials. Moreover, LDHs with different intercalated ions, sizes, and thicknesses have been utilized in the preparation of the LDHs-BPBA-PVA composite films, and the anions in the interlayer of LDHs showed a negligible effect on the phosphorescence performances for the composites (Supplementary Fig. 14). Similar results could be observed for the LDHs with the size of 50 nm prepared by colloidal milling (Supplementary Figs. 14 and 15). For the ultrathin LDH nanosheets, the phosphorescent intensities were slightly decreased for the composites, which could be induced by the existence of formamide to maintain the ultrathin morphology of the LDH nanosheets. These results suggested that the intercalated anions or the stacked layers of LDHs would not induce a significant influence on the LDHs-BPBA-PVA composites.

To validate the selectivity of the B‒O reaction, polymers without hydroxyl groups were employed in the construction of the composites, such as positively charged poly(diallyldimethyl-ammonium chloride) (PDDA) and negatively charged poly(styrene-4-sulfonate) (PSS). It could be observed that the phosphorescence intensities of the composites prepared by PDDA or PSS were only 36% and 23% of that prepared by PVA (Supplementary Fig. 16), ascribing to the absence of the B–O reaction for PDDA and PSS. Furthermore, PVA with the alcoholysis degrees of 98%, 92%, 87%, and 72% (denoted PVA98, PVA92, PVA87, and PVA72) were used to prepare the composite films. The phosphorescence and fluorescence intensities of 6% LDHs-BPBA-PVA composite films were reduced when the alcoholysis degrees of PVA decreased from 98% to 72% (Fig. 4d and Supplementary Fig. 17). After removing the UV lamp, the afterglow time of LDHs-BPBA-PVA92 and LDHs-BPBA-PVA87 was significantly shortened than that of LDHs-BPBA-PVA98, and LDHs-BPBA-PVA72 only showed a rapid afterglow of 2 s (Fig. 4e). This phenomenon could be further verified by the measurements of RTP lifetime (Fig. 4f): LDHs-BPBA-PVA98 showed an ultralong RTP lifetime up to 1.45 s, while the RTP lifetimes for LDHs-BPBA-PVA92, LDHs-BPBA-PVA87, and LDHs-BPBA-PVA72 were only 0.555 s, 0.293 s, and 0.153 s, respectively. The depressed RTP behaviors of LDHs-BPBA-PVA could be ascribed to the replacement of hydroxyl groups by the acetate groups in PVA with the lowered alcoholysis (Supplementary Fig. 10). Accordingly, the decreased contents of hydroxyl groups in PVA resulted in the reduced B‒O covalent bonds, manifested as the attenuated stretching vibration peak at 1310‒1430 cm^−1^ from FT-IR measurements (Supplementary Fig. 18). These results verified that multiple B‒O covalent bonds existed in the composite films to form a reticular cross-linking network between BPBA, PVA, and LDHs, and the phosphorescence intensities and lifetimes could be adjusted by tuning the quantities of hydroxyl groups in LDHs and PVA sophisticatedly. In conclusion, the more covalent bonds to construct the cross-linking network, the more efficient phosphorescence behaviors of the composite films could be realized.

### Mechanical property studies for the composite films

**Fig. 5:**
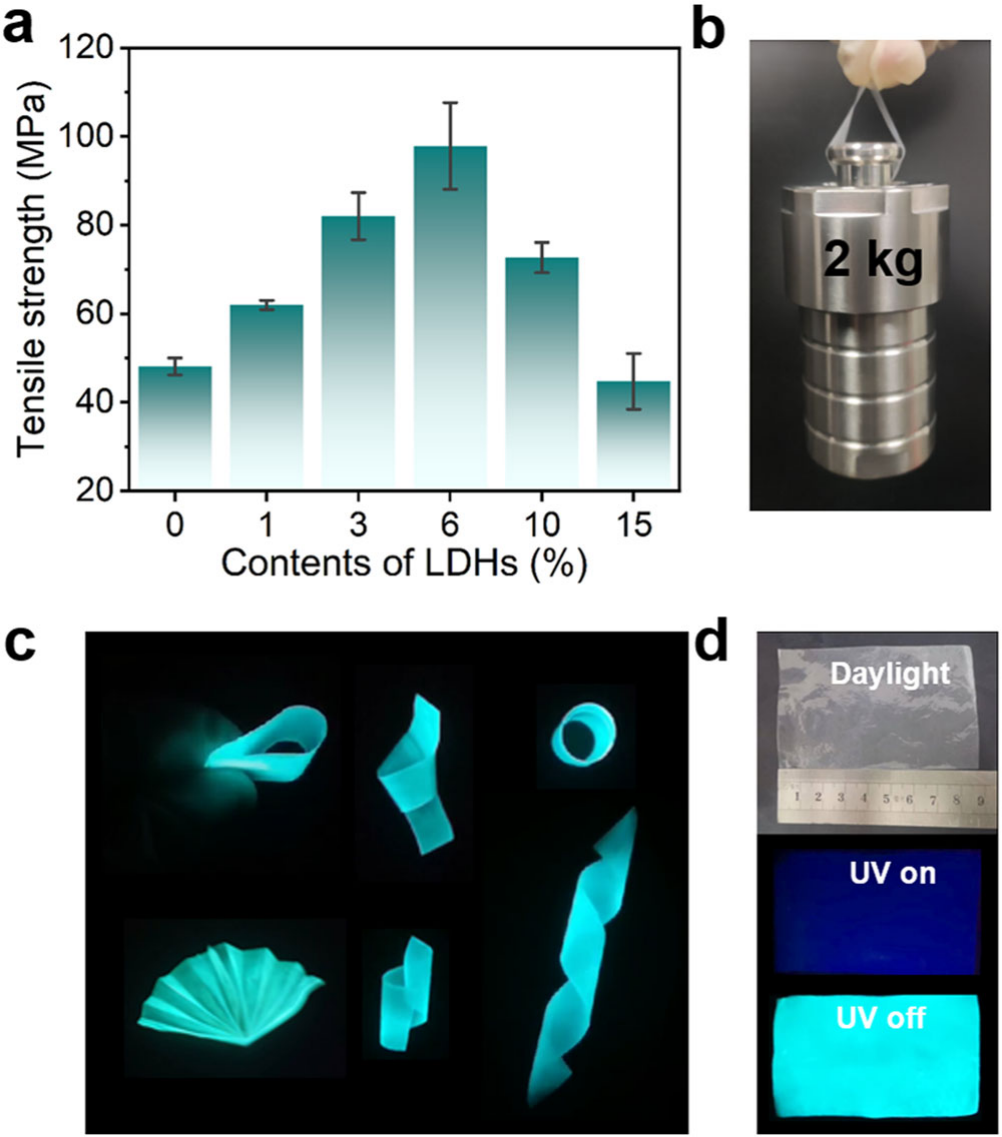
Mechanical properties of LDHs-BPBA-PVA composite films. **a** Tensile strengths of *x*% LDHs-BPBA-PVA composite films (the error bars represented the standard deviations from three parallel experiments). **b** Photograph of 6% LDHs-BPBA-PVA film lifting an object of 2 kg. Photographs of the LDHs-BPBA-PVA films (**c**) under deformations and (**d**) in a large-scale preparation.

The tensile strengths of all the composite films were implemented to evaluate the mechanical properties. The tensile strength of pure PVA was 45.2 MPa (Supplementary Table 3), which was comparable to the reported results^57,58^. Upon the introduction of BPBA, the 0% LDHs-BPBA-PVA film exhibited a tensile strength of 48.1 MPa. After the click reaction between LDHs, BPBA, and PVA, the mechanical properties of the *x*% LDHs-BPBA-PVA composites were significantly reinforced (Fig. 5a). Impressively, the tensile strength of 6% LDHs-BPBA-PVA reached 97.9 MPa, which was 2.17-fold higher than the pure PVA. Note that the tensile strength of 6% LDHs-PVA film was only 63.8 MPa, inferior to the 6% LDHs-BPBA-PVA film. The significantly promoted mechanical properties indicated that BPBA played a key role as a cross-linking agent to reinforce the interfacial interaction between organic PVA and inorganic LDHs phases. Moreover, the over-dose of LDHs (contents higher than 10%) might lead to the undesired aggregation of LDHs and the deteriorated mechanical properties of the composites^59,60^. It should be noted that the prominent phosphorescence and mechanical behaviors could be simultaneously achieved for the 6% LDHs-BPBA-PVA film, suggesting the effectiveness of the established cross-linking network. In addition, a small piece of 6% LDHs-BPBA-PVA film with a size of 8 × 1 cm^2^ and thickness of 0.1 mm was cut, and this film could be used to lift a 2 kg reactor without breaking (Fig. 5b). The composite films showed high tensile strength and promising applications for the optical devices.

Except for the high mechanical strength, luminescent polymeric materials should have good flexibility to meet the requirements for the applications as anti-counterfeiting devices, security labels and organic light-emitting diodes^61–65^. The LDHs-BPBA-PVA composite films were mechanically flexible and robust against mechanical deformations. These films could be bent, twisted and folded into round, fanshaped, heliciform, coiled and many other shapes, and bright cyan phosphorescence could be observed throughout the entire area (Fig. 5c). In the meanwhile, the films could be manufactured into scalable sizes (9 × 5.5 cm^2^ in Fig. 5d), maintaining their phosphorescence behaviors throughout the films. Such a mechanical-reliable, phosphorescent-robust and scalable film could find great potential in flexible 3D objects with repeatable folding and crimping properties on a large scale.

### Phosphorescence responses of the composite films towards mechanical deformation

**Fig. 6:**
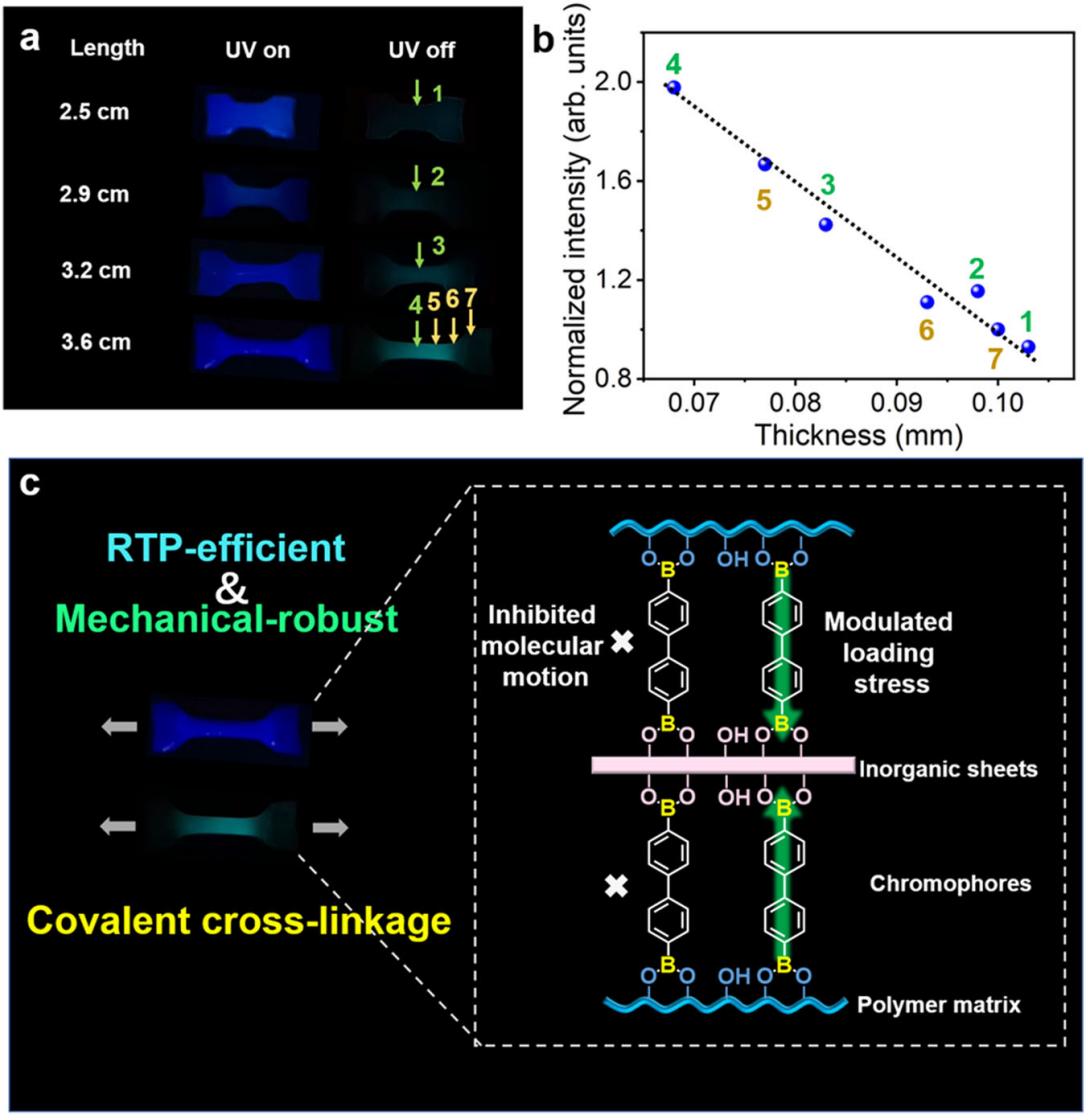
Mechanical responses and schematic representation of the 6% LDHs-BPBA-PVA films. **a** Fluorescence and delayed afterglow of dumbbell-shape specimen of LDHs-BPBA-PVA films under deformations; **b** Normalized RTP intensity variations for the films after stretching as a function of the thickness; **c** Schematic representation of the cross-linked composite films for the RTP responses towards mechanical deformations.

To explore the phosphorescence behaviors of the films under mechanical deformation, the LDHs-BPBA-PVA films with dumbbell-shape were prepared and then artificially stretched. The pristine film showed a length of 2.5 cm, with blue fluorescence and cyan phosphorescence in the presence and absence of UV light (Fig. 6a). When the LDHs-BPBA-PVA films were stretched from 2.5 cm to 3.6 cm, the deformation could be visualized by the brightened phosphorescence emissions of the deformed area in the films. Such a strain-responsive behavior of the LDHs-BPBA-PVA films were further confirmed by the phosphorescence spectra. The phosphorescence intensities of the neck regions in the films (annotated as positions 1–4) increased gradually when the films were stretched from 2.5 to 3.6 cm (Supplementary Fig. 19a), along with the decreased thickness from 0.100 mm to 0.068 mm measured by a high-precision thickness gauge with an accuracy of 0.001 mm. Note that the neck region (position 4) showed the brightest phosphorescence emissions than the shoulder (positions 5 and 6) and the bulk (position 7) regions (Supplementary Fig. 19b). The phosphorescence intensities of the films showed a linear proportion with the thickness of the film after stretched (Fig. 6b). In comparison, fluorescence responses with lower sensitivity could be acquired for the composite films under the same stretching experiment (Supplementary Fig. 20), which could be induced by the interference background of the fluorescence. The promoted mechanical properties with sensitive RTP responses of the composite films could be ascribed to the rigid organic-inorganic covalent network (Fig. 6c). When the external forces were applied, the stress on the *x*% LDHs-BPBA-PVA composite films could be tolerated through two approaches. First, the stress could be dissipated through the abundant covalent bonds in the cross-linking network^26^. On the other hand, the inorganic LDHs showed profound effect on bearing the stress, and the tensile load could be transferred from the polymer matrix to the rigid LDHs through the covalent bonds^18^. The modulated loading stress could affect the molecular arrangement and motion of the BPBA chromophores, leading to the RTP responses. Therefore, the luminescence properties of the composite films could also be utilized to understand the arrangement of the molecules and the structures of the covalently cross-linked network. When the boronic acid groups on the two sides of BPBA molecules reacted with LDHs and PVA, the BPBA molecules were immobilized in a covalently cross-linked network. Accordingly, the inhibited molecular motions of BPBA could lead to the decayed non-radiative transition and the boosted luminescence behaviors, and the loading stress of the composites could be dissipated through the bridged BPBA to the rigid inorganic sheets. It is believed that these observable phosphorescence signals could provide valuable opportunities for visual monitoring of material deformation and early damage reporting.

## Discussion

In summary, we have proposed the B–O click reaction between inorganic and organic components to establish a covalent cross-linking network for mechanical-robust and flexible polymer composites with the ultralong RTP performances. Such a rigid network could effectively weaken the non-radiative transition of chromophores for the boosted RTP performances, and the tensile loading stress could be dissipated through the covalent bonds to achieve a mechanical-robust composite film. These results demonstrated the power of the covalent bonds on the precise structural regulation and simultaneous enhancement of phosphorescence and mechanical properties for polymer composites. It is expected that the proposed strategy by the multiple covalent bonds could provide an ideal platform for developing the robust, durable and flexible RTP materials for the applications in the fields of anti-counterfeiting and information storage.

## Methods

### Materials

Analytical-grade chemicals, including K_2_CO_3_, KHCO_3_, dimethyl sulfoxide (DMSO), NaNO_3_, Mg(NO_3_)_2_•6H_2_O, Al(NO_3_)_3_•9H_2_O and urea were purchased from Sinopharm Chemical Reagent Co. Ltd. NaOH, HNO_3_, and Polyvinyl alcohol (PVA) with the different degrees of alcoholysis was obtained from Aladdin Chemical Co. Ltd.: PVA98 (98% hydrolyzed), PVA92 (92% hydrolyzed), PVA87 (87% hydrolyzed) and PVA72 (72% hydrolyzed). 4,4’-Biphenyldiboronic acid (BPBA) and sodium dodecyl sulfate (SDS) were purchased from J&K Scientific Ltd. Poly(diallyldimethyl-ammonium chloride) (PDDA) and poly(styrene-4-sulfonate) (PSS) was purchased from HWRK Co. Ltd., and formamide was purchased from J&K. Co. Ltd. All chemicals used in this experiment are analytical reagent, and all the chemicals are purchased and used directly without further purification.

### Preparation of the *x*% LDHs-BPBA-PVA and controlled films

PVA (3 g) was dissolved in 100 mL deionized water at 85 °C and stirred for 1 h. To hydrolyze the boronic acid of BPBA molecules, a buffer solution with pH of 10.5 was prepared by K_2_CO_3_ and KHCO_3_. BPBA (2 mg) was dissolved in DMSO and diluted by the buffer solution to get an alkaline solution (0.5 mg/mL). LDH suspensions with the different quantities from 5 mg, 15 mg, 30 mg, 50 mg, to 75 mg were added to 0.25 mL BPBA solution under stirring to acquire LDHs-BPBA composites. Afterwards, the PVA aqueous solution (16.7 mL) were added into the above mixture and stirred continually for 1 h. The as-prepared composites were poured into the plastic dish and dried in the oven at 60 °C for 4 h. Finally, the composite films were peeled off and labelled as *x*% LDHs-BPBA-PVA (*x*% stood for the weight ratio of LDHs to PVA on a dry basis, and it varied from 1 wt%, 3 wt%, 6 wt%, 10 wt% to 15 wt%). Note that the concentration of BPBA was maintained at 0.1 wt% for all the samples. The pure PVA film was prepared at 60 °C for 4 h, and the PVA-LDHs film was acquired with the LDH content of 6 wt%.

Similarly, the controlled experiments were carried out. Firstly, PVA with the different alcoholysis degrees of 98%, 92%, 87%, and 72% were employed (short as PVA98, PVA92, PVA87, and PVA72), and the composite films were prepared by BPBA (0.1 wt%), LDHs (6 wt%) and PVA. Secondly, LDH nanosheets with the tunable hydroxyl groups were obtained by adding the different quantities of sodium dodecyl sulfate (SDS, 7.5 mg, 15 mg, and 30 mg) into the LDH suspensions (30 mg), followed by the ultrasonic treatment for 1 h. Then the SDS@LDHs-BPBA-PVA composite films were prepared according to the above procedures (BPBA of 0.1 wt% and LDHs of 6 wt%). Similarly, the LDHs-BPBA-PDDA and LDHs-BPBA-PSS were prepared according to these procedures.

### Sample characterization

XRD of all the films and LDH particles were measured on a Rigaku 2500 VB2 + PC diffractometer in the range from 5° to 60°. The FT-IR spectra of the films were measured on a Nicolet 6700 (Thermo Electron) in the range of 4000‒600 cm^−1^. The photos of the phosphorescence for the composites were captured after irradiation under a 280 nm UV LED module for 3 s. The fluorescence and phosphorescence spectra of all the samples were recorded using an F-7000 spectrophotometer (Hitachi, Japan) with an excitation wavelength of 280 nm. The fluorescence emission spectra were collected in the range of 350‒650 nm, and the voltage of the photomultiplier tube was set at 300 V. The phosphorescence spectra were collected ranging from 400–600 nm with the voltage of 500 V. Fluorescence and phosphorescence lifetime measurements were implemented on an Edinburgh Instruments FLS 980 fluorimeter. The fluorescence quantum yields were measured on FS5 fluorescence spectrometer. The tensile strengths of the as-prepared films were performed using the testing machine CMT4104 (MTS, China) with an extension rate of 50 mm/min.

### Supplementary information

Supplementary Information

Peer Review File

### Source data

Source Data

## Data Availability

Data supporting the findings of this manuscript are available from the corresponding author upon request. Source data are provided with this paper.
