# Supplementary material for: Design of mechanical-robust phosphorescence materials through covalent click reaction
Source: Nat Commun. 2023 Aug 5;14:4720. doi: 10.1038/s41467-023-40451-2 (PMC10404264; doi:10.1038/s41467-023-40451-2)
Supplement: Supplementary file 1 — Supplementary Information [file 41467_2023_40451_MOESM1_ESM.pdf]

Supplementary Information

Design of mechanical-robust phosphorescence materials through covalent click reaction

Rui Tian,*[1] Shuo Gao,[1] Kaitao Li,[1] and Chao Lu*[1,2]

[1]*State Key Laboratory of Chemical Resource Engineering, Beijing University of Chemical Technology, North Third Ring Road 15, Chaoyang District, Beijing, China*

[2]*Green Catalysis Center, College of Chemistry, Zhengzhou University, No.100 Science Avenue, Zhengzhou, China*

Fax/Tel.: +86 10 64411957. E−mail: tianrui@mail.buct.edu.cn; luchao@mail.buct.edu.cn.

CONTENTS

1. Supplementary Methods....S4

Preparation of MgAl-LDHs S4

Sample characterization S4

2. Supplementary Figures....S4

Figure S1. Molecular formulae of PVA, BPBA and LDH. S5

Figure S2. SEM and TEM images of MgAl-LDHs.. ....S6

Figure S3. XRD patterns and FTIR spectra for *x*%LDHs-BPBA-PVA films. S7

Figure S4. XPS spectra for BPBA and 6% LDHs-BPBA-PVA film....S8

Figure S5. Element analysis and EDS mapping of 6%LDHs-BPBA-PVA films....S9

Figure S6. Fluorescence and phosphorescence intensities of LDHs and LDHs-BPBA S10

Figure S7. Fluorescence spectra of *x*%LDHs-BPBA-PVA films....S11

Figure S8. Fluorescence lifetime measurements of BPBA and of *x*%LDHs-BPBA-PVA films S12

Figure S9. UV-Vis absorption spectra of 6%LDHs-BPBA-PVA film and controlled samples S13

Figure S10. Schematic representation of the regulation of the B–O covalent bonds S14

Figure S11. Zeta potential measurements for LDHs, SDS and LDH@SDS S15

Figure S12. Fluorescent spectra of 6%LDHs-BPBA-PVA films modified with SDS S16

Figure S13. XRD patterns of 6%LDHs-BPBA-PVA films modified with SDS S17

Figure S14. Phosphorescent spectra of composite films prepared by differrent LDHs....S18

Figure S15. TEM image of LDHs prepared by colloidal milling S19

Figure S16. Phosphorescence spectra of composites prepared by different polymers. S20

Figure S17. Fluorescence spectra of composites prepared by PVA with different alcoholysis degree S21

Figure S18. FTIR spectra of composites prepared by PVA with different alcoholysis degree S22

Figure S19. Phosphorescence spectra of 6%LDHs-BPBA-PVA films stretched to the different lengths S23

Figure S20. Fluorescent spectra of 6%LDHs-BPBA-PVA films stretched to the different lengths S24

3. Supplementary Tables S25

Table S1. Φ_{fluo}, τ_{Fluo}, and τ_{Phos} for BPBA and *x*%LDHs-BPBA-PVA composites S25

Table S2. Dynamic photophysical parameters of *x*%LDHs-BPBA-PVA composites S26

Table S3. Mechanical properties of PVA, LDHs-PVA and *x*%LDHs-BPBA-PVA films ... S27

4. Supplementary Reference S28

1. Supplementary Methods

Preparation of MgAl-LDHs. To prepare MgAl-LDHs intercalated with CO_3^{2-}, $Mg(NO_3)_2{\bullet}6H_2O$ (0.02 mol), $Al(NO_3)_3{\bullet}9H_2O$ (0.01 mol) and urea (0.12 mol) were dissolved in 80 mL deionized water, and the suspension was hydrothermally treated in an autoclave at 100 °C for 24 h.[1] The resulting slurry was centrifuged and washed for three times with deionized water. Finally, the LDH suspensions were quantified by the inductively coupled plasma-atomic emission spectrometry (ICP-AES) and left for use. MgAl-LDHs intercalated with NO_3^- was prepared afterwards through an ion-exchange process in a solution containing $NaNO_3$ (1.7 mol/L) and HNO_3 (5.3 mmol/L) for 36 h. Ultrathin LDH nanosheets were exfoliated from MgAl-LDHs with NO_3^- in the formamide under room temperature for 48 h. MgAl-LDHs with the size of ~50 nm was prepared by colloidal milling with the raw materials of $Mg(NO_3)_2{\bullet}6H_2O$ (0.2 mol/L), $Al(NO_3)_2{\bullet}9H_2O$ (0.1 mol/L) and NaOH (3 mol/L).

Sample characterization. The morphologies of the films were examined using SEM (JEOL JSM-7800F) in combination with EDS, and the morphologies of LDHs were studied by SEM and transmission electron microscopy (TEM, Hitachi HT7700). X-ray photoelectron spectroscopy (XPS) measurements were recorded using an ESCALAB 250 instrument (Thermo Electron) with Al Kα radiation. UV-vis absorption spectra of the composites were recorded using a Shimadzu UV-3600 spectrophotometer (Japan). Zeta potential measurements of the samples were performed on Light Scattering Particle Size and Zeta potentiometer (ZS90). The elemental compositions of LDHs were performed on inductively coupled plasma (ICP) emission spectroscopy (Shimadzu ICPS-7500).

2. Supplementary Figures

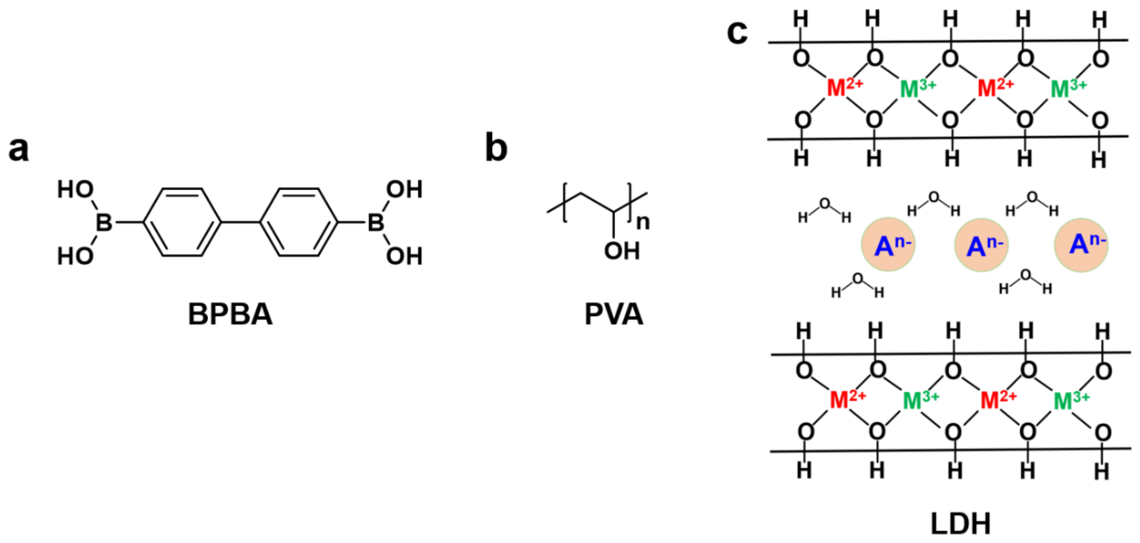

Supplementary Figure 1. Molecular formulae of (**a**) 4,4'-biphenyldiboronic acid (BPBA) molecules, (**b**) polyvinyl alcohol (PVA), and (**c**) layered double hydroxide (LDHs, M^{2+}/M^{3+} stand for the divalent/trivalent metal ions in the host layer and A^{n-} represents the anion in the interlayer of LDHs).

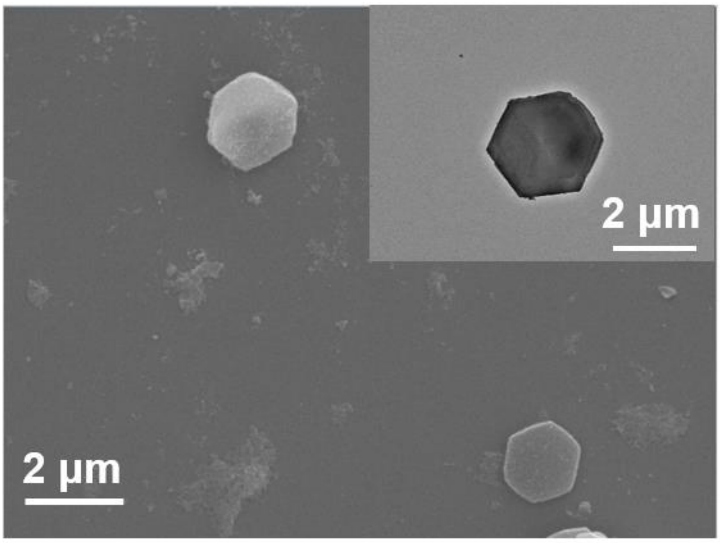

Supplementary Figure 2. SEM and TEM (inset) images of MgAl-LDHs.

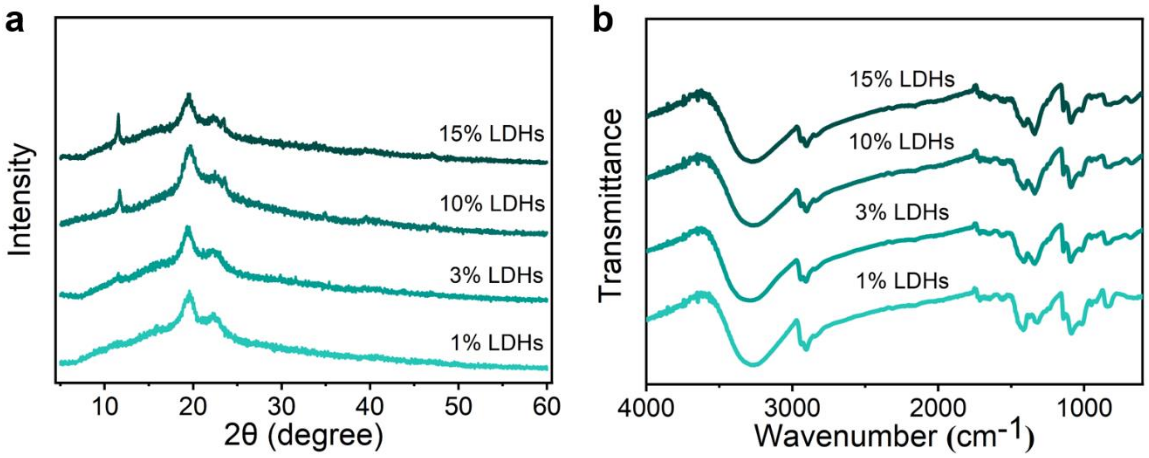

Supplementary Figure 3. a XRD patterns and **(b)** FTIR spectra for *x*%LDHs-BPBA-PVA films with the contents of LDHs varied from 1 wt % to 15 wt% of PVA.

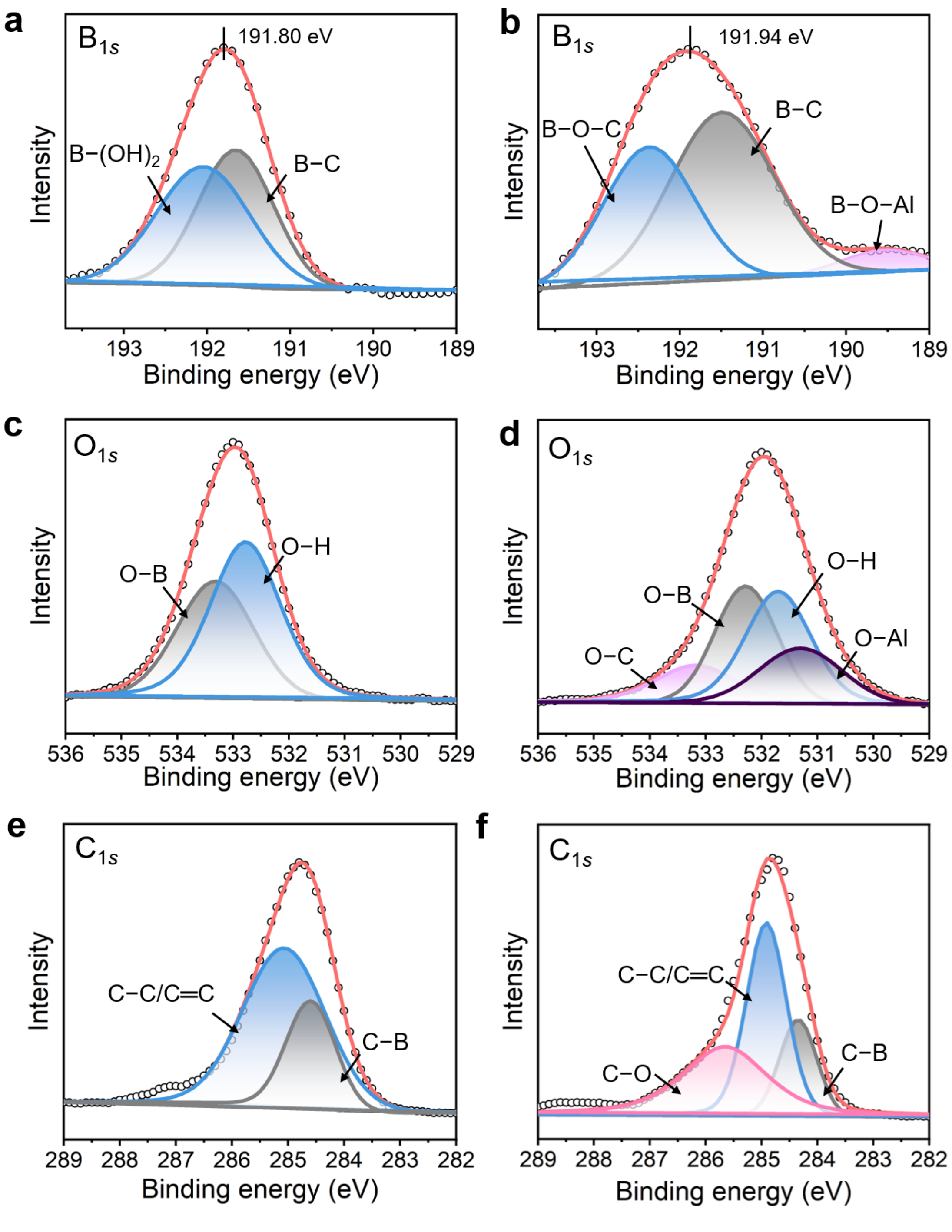

Supplementary Figure 4. XPS spectra of (**a** and **b**) B 1*s*, (**c** and **d**) O 1*s*, and (**e** and **f**) C 1*s* for (**a**, **c**, **e**) BPBA and (**b**, **d**, **f**) 6% LDHs-BPBA-PVA film.

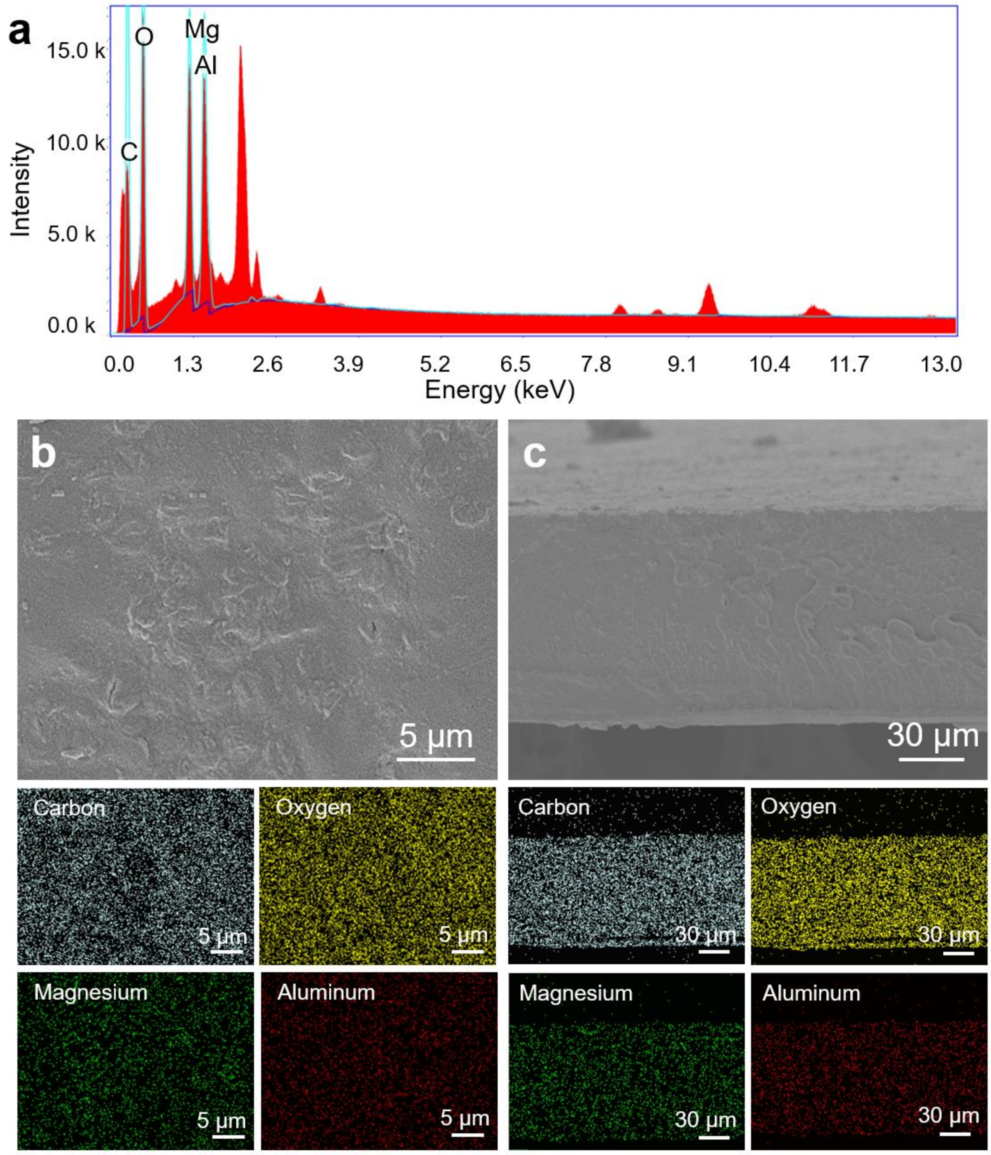

Supplementary Figure 5. a Element analysis, EDS mapping of carbon, oxygen, magnesium and aluminum elements for 6%LDHs-BPBA-PVA films from (**b**) top-view and (**c**) side-view images.

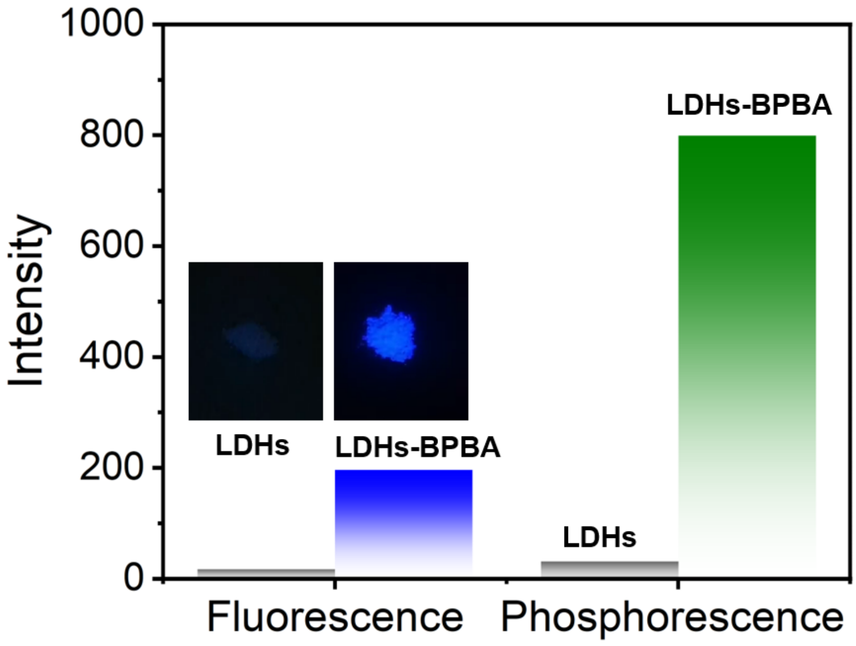

Supplementary Figure 6. Fluorescence and phosphorescence intensities of LDHs and LDHs-BPBA, and the inset showed the fluorescent photos of LDHs and LDHs-BPBA under UV light.

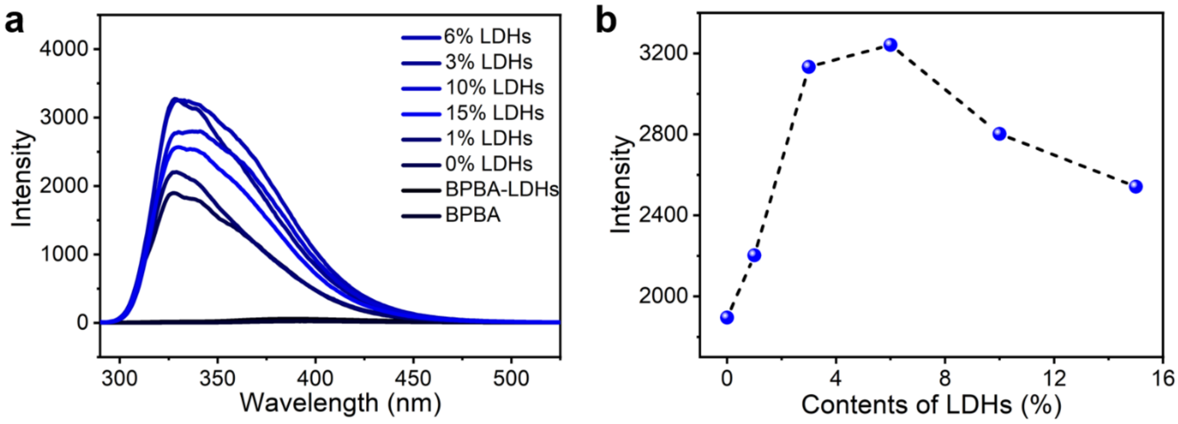

Supplementary Figure 7. a Fluorescence spectra of *x*%LDHs-BPBA-PVA films with the contents of LDHs varied from 0 wt % to 15 wt% of PVA; **b** Fluorescence intensity variations of *x*%LDHs-BPBA-PVA films.

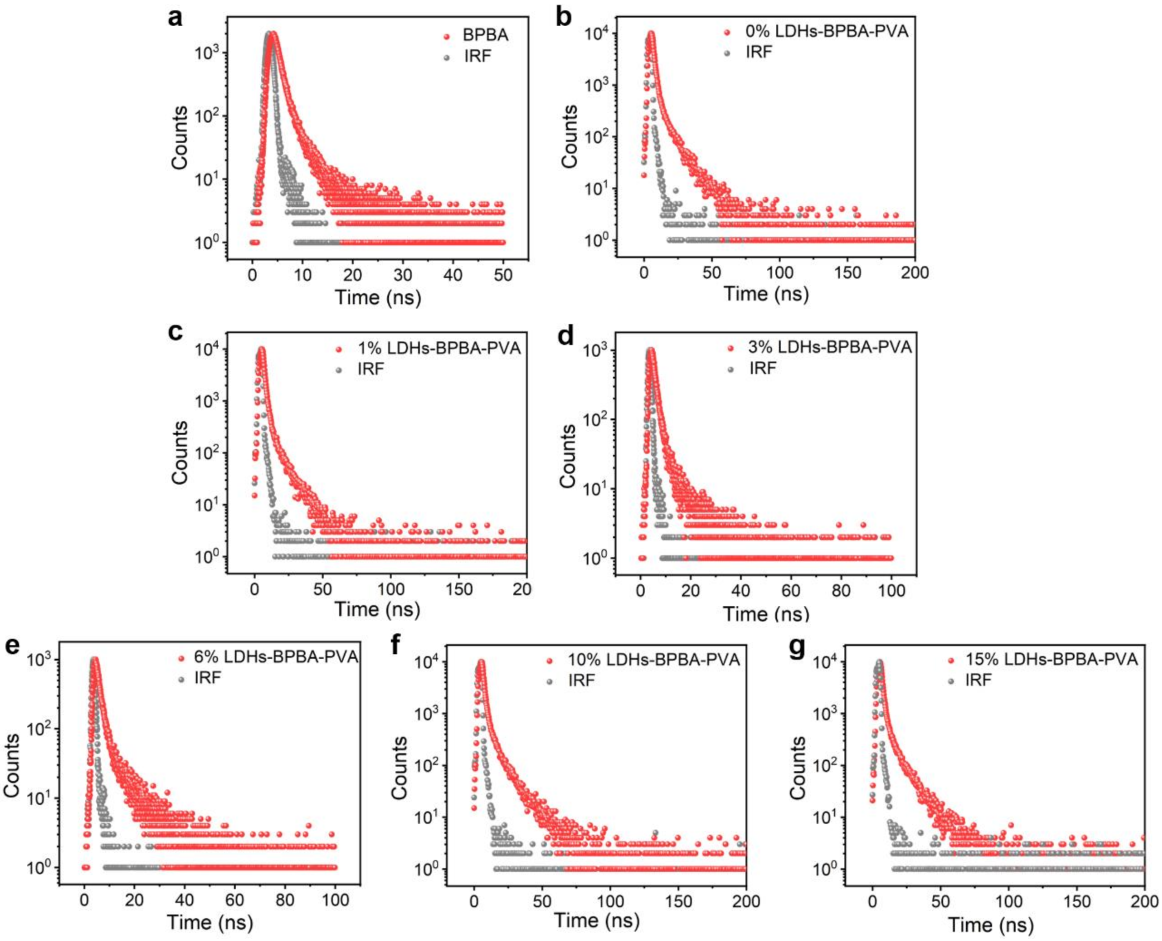

Supplementary Figure 8. Fluorescence lifetime measurements for (**a**) BPBA, (**b**) BPBA-PVA and *x*%LDHs-BPBA-PVA for (**c**) *x*= 1, (**d**) *x*=3, (**e**) *x*=6, (**f**) *x*=10, and (**g**) *x*=15.

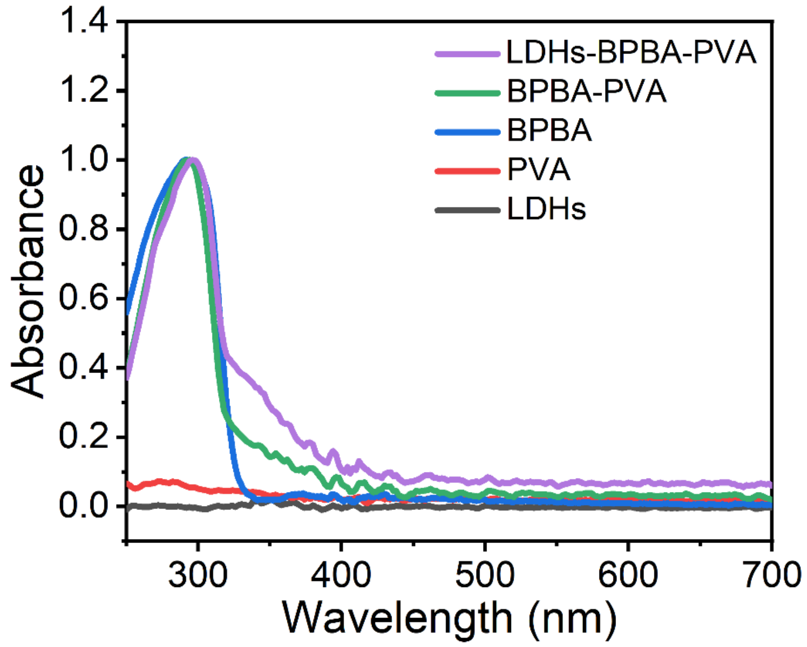

Supplementary Figure 9. Normalized UV-Vis absorption spectra of 6%LDHs-BPBA-PVA film and controlled samples.

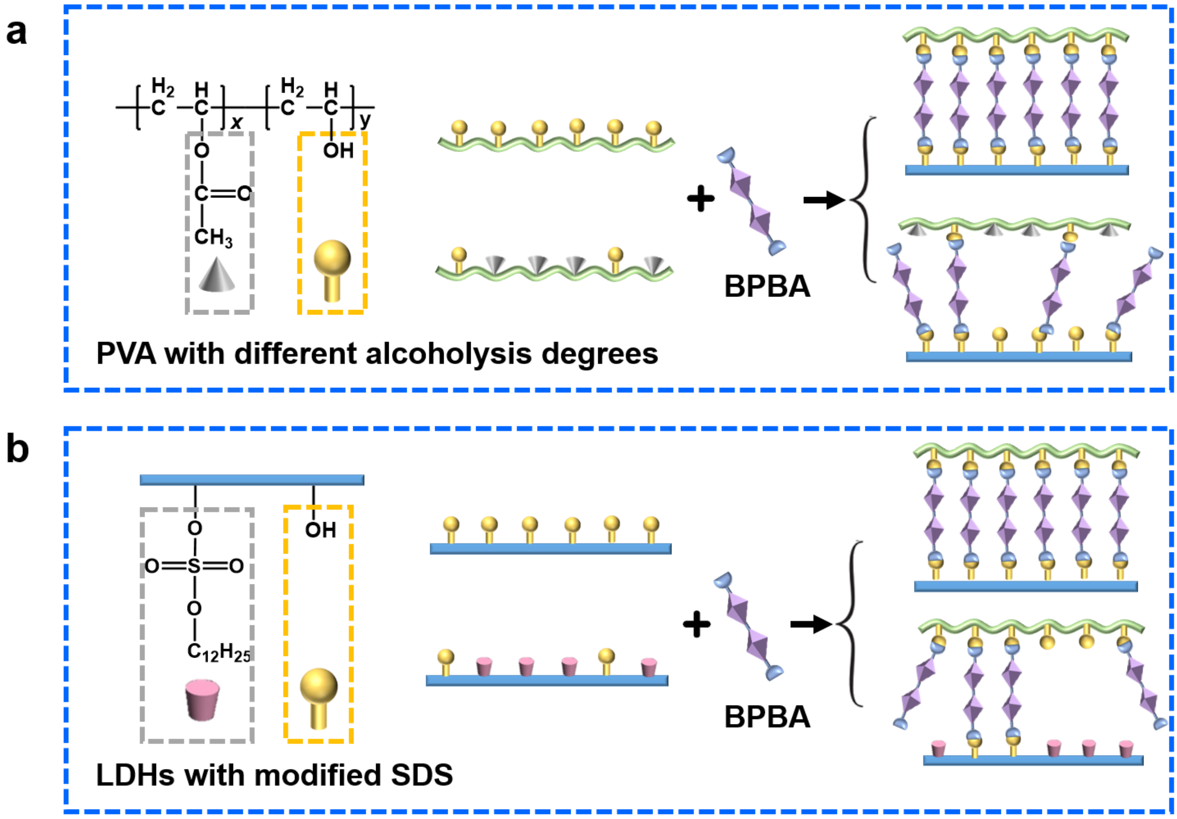

Supplementary Figure 10. Schematic representation of the regulation of the B–O covalent bonds based on the adjustment of (**a**) PVA and (**b**) LDHs.

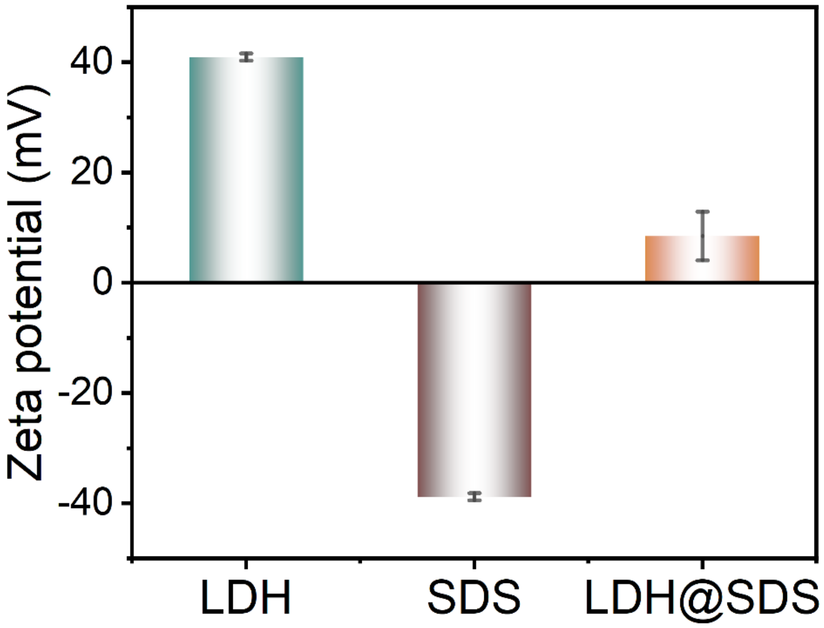

Supplementary Figure 11. Zeta potential measurements for LDH, SDS and LDH@SDS.

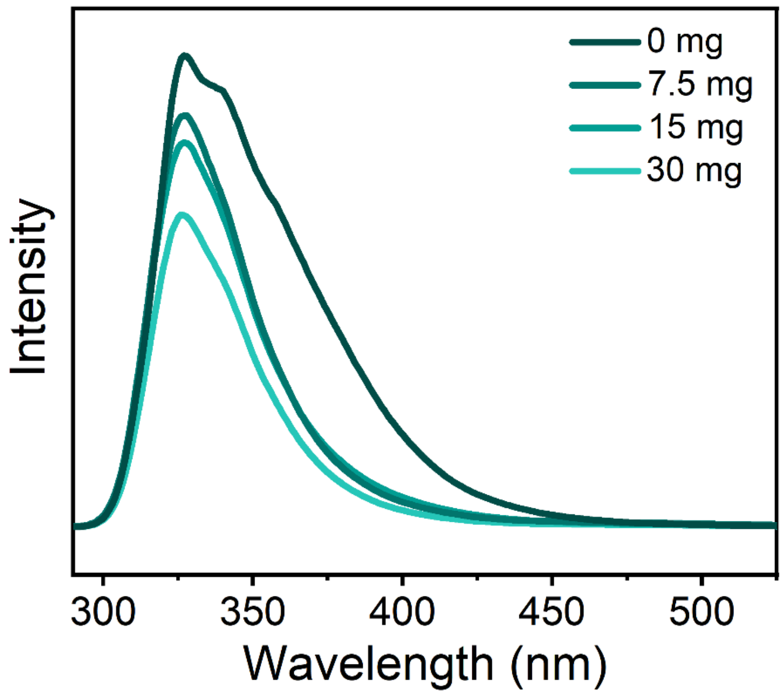

Supplementary Figure 12. Normalized fluorescent emission spectra of 6%LDHs-BPBA-PVA films for LDHs modified with different quantities of SDS.

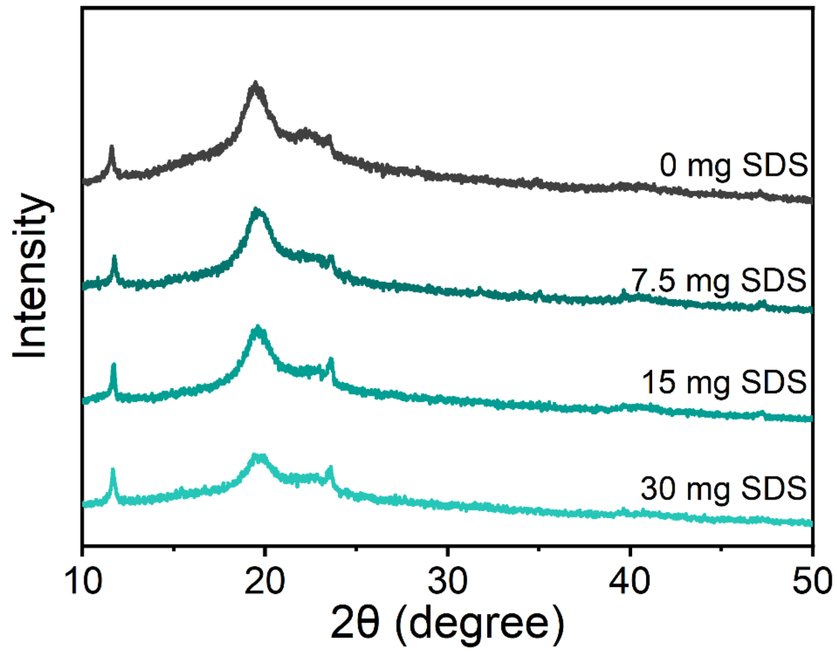

Supplementary Figure 13. XRD patterns of 6%LDHs-BPBA-PVA films for LDHs modified with different quantities of SDS.

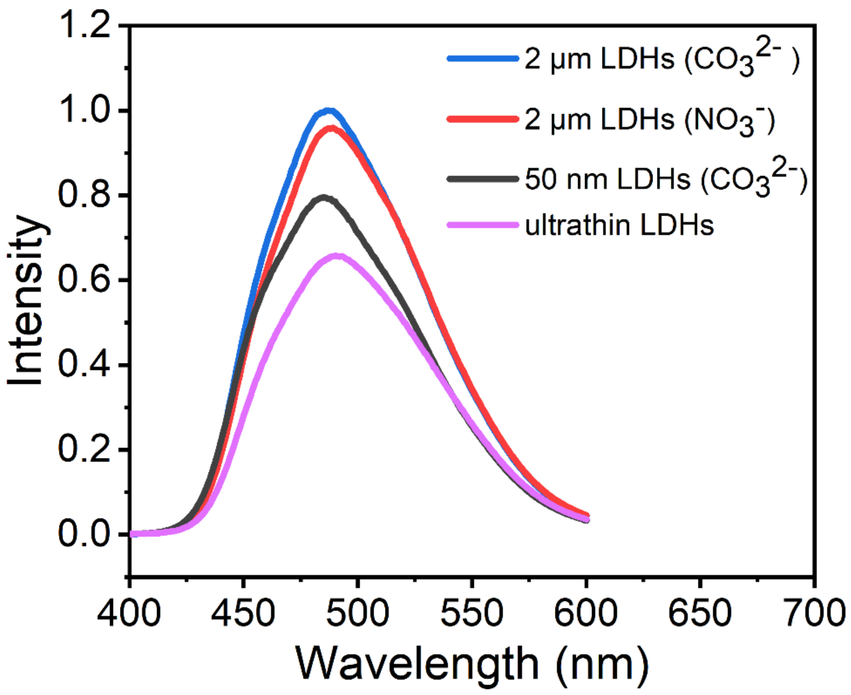

Supplementary Figure 14. Normalized phosphorescent emission spectra of LDHs-BPBA-PVA composites prepared by LDHs with different sizes and intercalated anions.

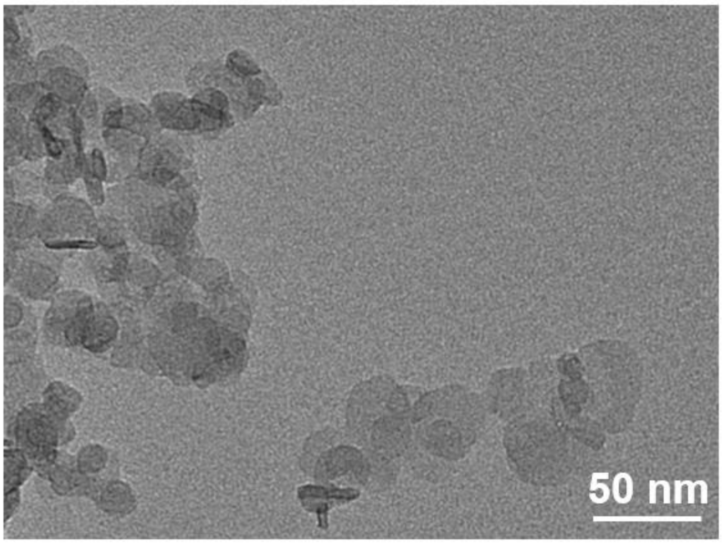

Supplementary Figure 15. TEM image of LDHs prepared by colloidal milling (50 nm CO_3^{2-}).

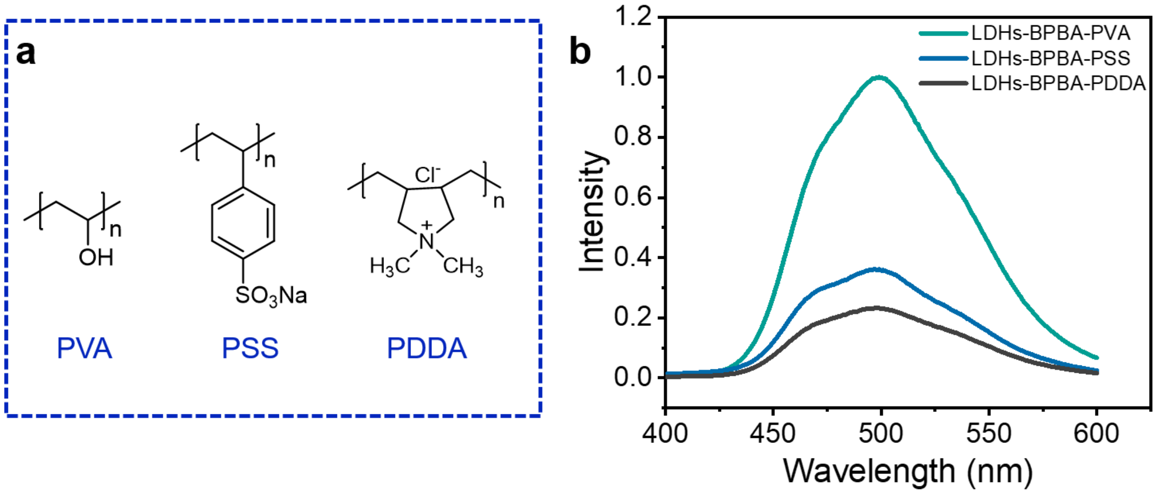

Supplementary Figure 16. a Molecular structure of PVA, poly(styrene-4-sulfonate) (PSS) and poly(diallyldimethyl-ammonium chloride) (PDDA), and (**b**) phosphorescence spectra of composites prepared by different polymers.

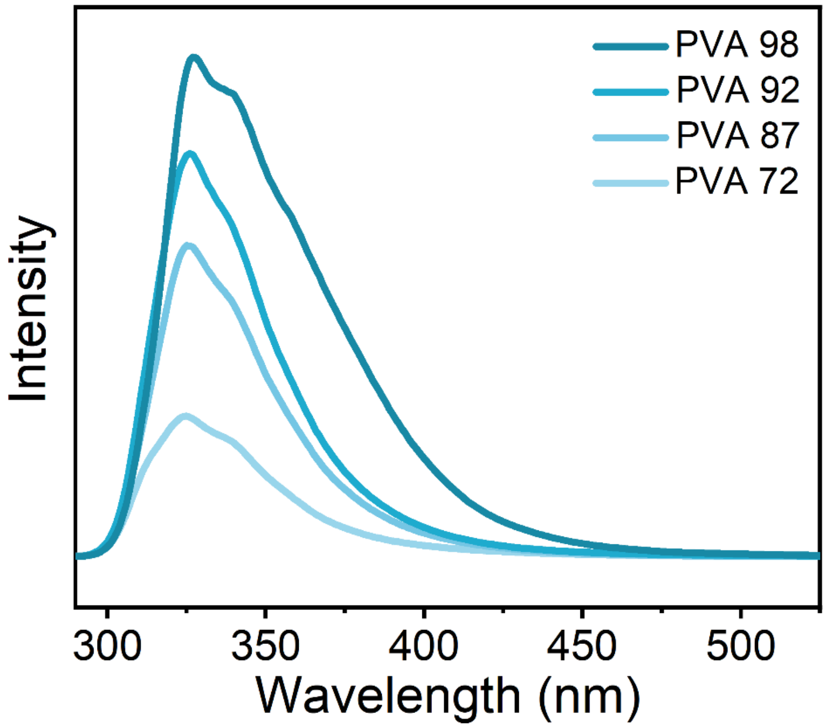

Supplementary Figure 17. Normalized fluorescent emission spectra of 6%LDHs-BPBA-PVA films for PVA with the alcoholysis degree ranged from 72%, 87%, 92% to 98%.

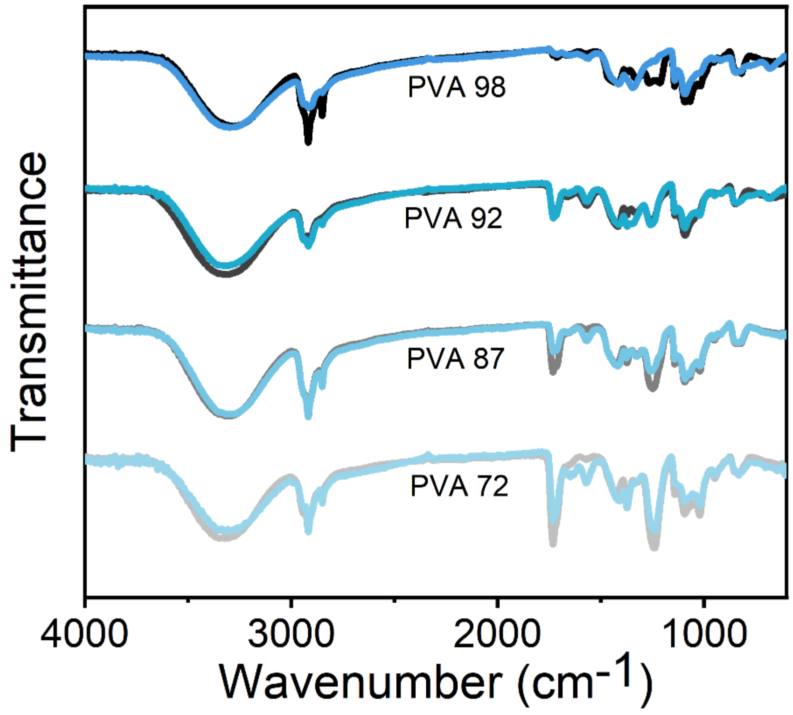

Supplementary Figure 18. FTIR spectra of 6%LDHs-BPBA-PVA (blue lines) and PVA (grey lines) with the alcoholysis degree of PVA ranging from 72%, 87%, 92% to 98%.

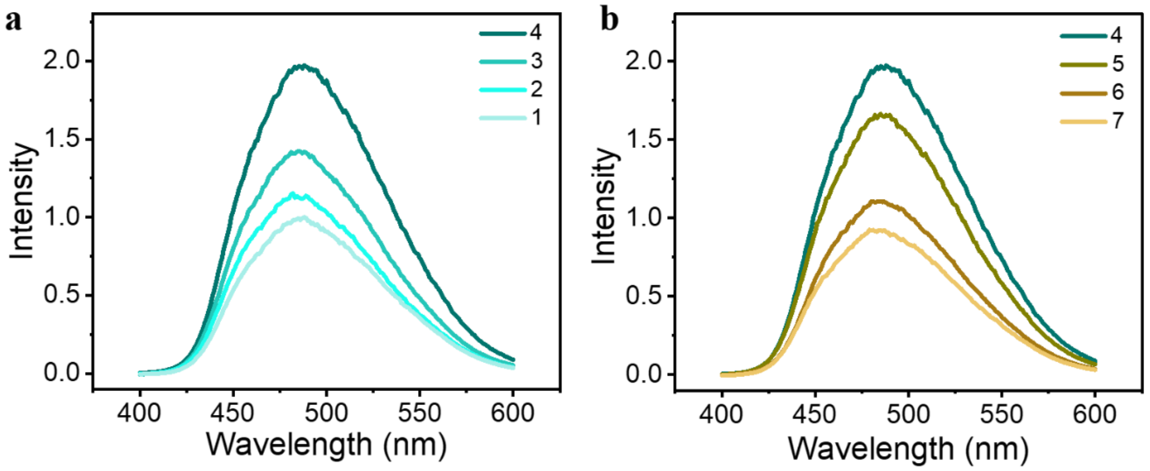

Supplementary Figure 19. Normalized phosphorescence emission spectra of 6%LDHs-BPBA-PVA films stretched to the different lengths at the different positions: **(a)** positions 1–4 and **(b)** positions 4–7.

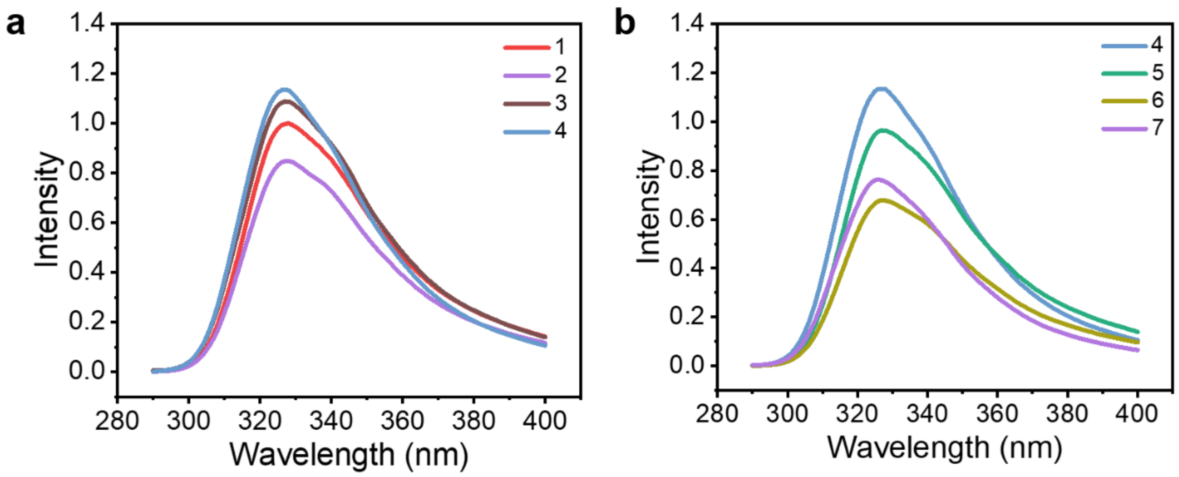

Supplementary Figure 20. Normalized fluorescent emission spectra of 6%LDHs-BPBA-PVA films stretched to the different lengths at the different positions: **(a)** positions 1–4 and **(b)** positions 4–7.

3. Supplementary Tables

Supplementary Table 1. The values of Φ_{fluo}, τ_{Fluo}, and τ_{Phos} for BPBA and *x*%LDHs-BPBA-PVA films (the contents of LDHs varied from 0 wt % to 15 wt%).

Sample	Φ_{fluo}/%	τ_{Fluo}/ns	τ_{Phos}/s
BPBA	2.94	1.29	0.007
BPBA-PVA	29.11	2.61	0.899
1%LDHs-BPBA-PVA	37.98	2.20	1.31
3%LDHs-BPBA-PVA	40.03	2.48	1.34
6%LDHs-BPBA-PVA	44.41	2.80	1.45
10%LDHs-BPBA-PVA	44.59	2.84	1.35
15%LDHs-BPBA-PVA	43.04	2.80	1.23

Supplementary Table 2. Dynamic photophysical parameters of BPBA-PVA and 6%LDHs-BPBA-PVA films.

	$\boldsymbol{k}_{\mathbf{isc}}$ /s^{-1}	$\boldsymbol{k}_{\mathbf{p,\ r}}$ /s^{-1}	$\boldsymbol{k}_{\mathbf{p,\ nr}}$ /s^{-1}
6%LDHs-BPBA-PVA	1.40×10^{7}	4.9×10^{-2}	0.64
BPBA-PVA	1.03×10^{7}	4.2×10^{-2}	1.07

The dynamic photophysical parameters were calculated based on the equations below:

$F_{\mathrm{isc}} = 1 - F_{\mathrm{f}} - F_{\mathrm{ic}} \approx 1 - F_{\mathrm{f}}$

$k_{\mathrm{p,\ r}} = F_{\mathrm{p}}/(F_{\mathrm{isc}} \times \tau_{\mathrm{p}})$

$k_{\mathrm{p,\ nr}} = 1/\tau_{\mathrm{p}} - k_{\mathrm{p,\ r}}$

$k_{\mathrm{isc}} = F_{\mathrm{p}}/\tau_{\mathrm{f}}$,

where τ_{p}, F_{p}, τ_{f}, and F_{f} stood for the lifetimes and quantum yields for the phosphorescence and fluorescence emissions, $k_{\mathrm{p,\ r}}$, $k_{\mathrm{p,\ nr}}$, and k_{isc} represented the rate constants for the radiative transition, non-radiative transition of phosphorescence and intersystem crossing (ISC).[2-4]

Supplementary Table 3. Mechanical properties of pure PVA, LDHs-PVA, and *x*%LDHs-BPBA-PVA films (the contents of LDHs varied from 0 wt % to 15 wt%).

**Sample**	**Contents of LDHs (wt%)**	**Tensile strength (MPa)**
Pure PVA	0	45.2 ± 3.8
LDHs-PVA	6	63.8 ± 2.4
x%LDHs-BPBA-PVA	0	48.1 ± 1.9
	1	62.0 ± 1.0
	3	82.0 ± 5.3
	6	97.9 ± 9.8
	10	72.7 ± 3.4
	15	44.7 ± 6.3

4. Supplementary Reference

1. Yu, J., Wang, Q., O'Hare, D. & Sun, L. Preparation of two dimensional layered double hydroxide nanosheets and their applications. *Chem. Soc. Rev.* **46**, 5950-5974, (2017).
2. Li, D. et al. Completely aqueous processable stimulus responsive organic room temperature phosphorescence materials with tunable afterglow color. *Nat. Commun.* **13**, 347, (2022).
3. Li, D., Yang, J., Fang, M., Tang, B. Z. & Li, Z. Stimulus-responsive room temperature phosphorescence materials with full-color tunability from pure organic amorphous polymers. *Sci. Adv.* **8**, eabl8392, (2022).
4. Xu, W. W. et al. Tunable Second-Level Room-Temperature Phosphorescence of Solid Supramolecules between Acrylamide-Phenylpyridium Copolymers and Cucurbit[7]uril. *Angew. Chem. Int. Ed.* **61**, e202115265, (2022).
