# Supplementary material for: Design of mechanical-robust phosphorescence materials through covalent click reaction
Source: Nat Commun. 2023 Aug 5;14:4720. doi: 10.1038/s41467-023-40451-2 (PMC10404264; doi:10.1038/s41467-023-40451-2)
Supplement: Supplementary file 2 — Peer Review File [file 41467_2023_40451_MOESM2_ESM.pdf]

nature portfolio

Peer Review File

Design of mechanical-robust phosphorescence materials through covalent click reaction

Open Access This file is licensed under a Creative Commons Attribution 4.0 International License, which permits use, sharing, adaptation, distribution and reproduction in any medium or format, as long as you give appropriate credit to the original author(s) and the source, provide a link to the Creative Commons license, and indicate if changes were made. In the cases where the authors are anonymous, such as is the case for the reports of anonymous peer reviewers, author attribution should be to 'Anonymous Referee' followed by a clear attribution to the source work. The images or other third party material in this file are included in the article's Creative Commons license, unless indicated otherwise in a credit line to the material. If material is not included in the article's Creative Commons license and your intended use is not permitted by statutory regulation or exceeds the permitted use, you will need to obtain permission directly from the copyright holder. To view a copy of this license, visit http://creativecommons.org/licenses/by/4.0/.

Reviewers' Comments:

Reviewer #1:
Remarks to the Author:
This manuscript describes the design and preparation of mechanical-robust room temperature phosphorescence materials through covalent click reaction. In this paper, the PVA-LDH composite has tensile strength of 97.9 MPa, 2.17-fold higher than pure PVA. There are many polymers and polymer composites with tensile strength higher than 100 MPa and even higher. It may be helpful to provide reasons for choosing PVA. It is known that PVA matrix has been frequently used by Y. Zhao and coworkers to fabricate polymer-based room-temperature phosphorescence materials. Other research group also use PVA to serve as matrix. Recently, Z. Yang and coworkers reported the preparation of triphenylene-2-ylboronic acid-PVA phosphorescence materials with lifetime above 3.0 s (Adv. Mater. 2022, 34, 2108333). In this paper, boronic acid has also been used as phosphorescence dopant, whereas the phosphorescence lifetime is moderate (1.45 s, when compared to other boronic acid system, such as Adv. Mater. 2022, 34, 2108333 and Angew. Chem. Int. Ed.) and the phosphorescence quantum yield (3.92%) is low. It is understandable that BPBA molecules with two boronic acid groups may serve as crosslinker for material fabrication. The statement of "covalent click reaction" need further evidence, for example, whether the reaction between boronic acid and other components exhibits the features of click chemistry in model system or this material system. There are molecules with two or more boronic acid groups from commercial source or by chemical synthesis, among which there may be efficient and long-lived RTP dopants. In the reported studies, there are other polymer-based RTP and afterglow materials, responsive RTP materials, and materials with other afterglow mechanisms; some representative studies or review articles can be cited. In many polymer-based RTP systems, the RTP properties can only be obtained when the materials are sufficiently irradiated by excitation sources, because of the present of oxygen under ambient conditions. For the composite materials in this paper, it may be better to provide such information about RTP activation in the main text. Overall, the design strategy of RTP materials in this paper is important, whereas the selection of matrix and dopant for high-performance RTP materials, and the demonstration of covalent click reaction need major revision.

Reviewer #2:
Remarks to the Author:
[Note from the editor: Please see attached PDF]

The authors in this manuscript reported the design and construction of room temperature phosphorescence (RTP) with decent mechanical properties, based on the click reaction between chromophores, inorganic particle and polymer. The as-prepared composites showed decent RTP performance with lifetime to 1.45 s and simultaneously promoted mechanical strength of 97.9 MPa. These promotions could be ascribed to the established cross-linkage and covalent-bond-accommodated interfacial interaction in the polymeric composites. The proposed construction strategy could be applied in scalable preparation of RTP materials. Therefore, I recommend this work to be published in Nature Communications after the following minor revisions.

1. The authors have shown the significant effect of layered double hydroxides (LDHs) in the construction of RTP-efficient and mechanical-robust composites. To better illustrate this idea, the basic information of LDHs, such as the structure, composition and morphology, should be supplemented. What is the structural formula of LDHs? How are the hydroxyl groups arranged in the LDHs? I do think such an introduction is beneficial to understand the design strategy and the decent performances of the composites.

2. To regulate the B-O covalent bonds, the authors have introduced sodium dodecyl sulfate (SDS) to modify the surface of LDHs. How could the SDS occupy the hydroxyl groups in LDHs? What is the interaction between SDS and LDHs?

3. The authors have introduced SDS to adjust the hydroxyl groups in LDHs and changed the alcoholysis degrees to regulate the hydroxyl groups in PVA in the "Regulation of the B−O covalent bonds" section. I recommend the authors to supplement the schematic representation for these regulations, so that the importance of the cross-linkage could be better understood.

4. The fluorescence lifetime measurements for the composites should be provided in the supplementary material to support the data in the Table S1.

5. How to measure the thickness of the films as shown in Fig. 6b?

6. As shown in Fig. 6a, the composite films also exhibited fluorescence responses towards mechanical deformations. How is the sensitivity of these fluorescence responses? Could the authors explain the possible reason for the different response sensitivity between fluorescence and phosphorescence?

7. To arouse a broad interest from readers in this field, some related works on polymer hybrid RTP and wide applications (Chem. Sci. 2016, 7, 4519; Chem. Soc. Rev., 2021, 50, 5564; Chem. Eng. J. 2022, 430, 133084) could to be enriched as references.

Reviewer #3:
Remarks to the Author:
The manuscript intended to be published at Nature Communication present a strategy to obtain a mechanical robust and flexible polymer composite based on the so-called B–O click reaction between inorganic and organic components.

Before the manuscript becomes suitable for publication in such prestigious journal, I kindly request the authors to address the following and, in point of view, important questions:

1. In the title, the authors use the term “Design of mechanical-robust room temperature phosphorescence materials”. I my opinion, the title in its current form can mislead the readers towards understanding that the material is prepared at room temperature not the emission of the chromophores.

2. Concerning the LDHs, it is not clear for me which type was used. Was it obtained commercially? Who are the metals in the structure?

3. The authors present the XRD pattern of the LDH showing some characteristic peaks. Indeed the 11.9° position correspond to the (003) crystal plane. What is the basal spacing associated to that? Who is the anionic molecule intercalated in this LDH?

4. I found strange using the fact of the appearance of the 11.9° peak in the XRD pattern sufficient for the authors to state that the introduction of LDHs into the composite films was successful obtained. I there another way to show that?

5. Would be possible to apply a complementary experiment to confirm the so-called B-O click, meaning it there a way to study the coordination/environment of the B?

6. Considering that LDH is a layered material, it is not clear for me where exact in the 4,4’-biphenyldiboronic acid (BPBA) molecules in that structure. Is it only in the external surface of the LDH? Choosing a different LDH with altered intercalated molecule would interfere in the process? Is the stacking number of Layers could also play a role?

7. The authors state “luminescent polymeric materials should have good flexibility to meet the requirements for the applications”. Which kind applications the authors have in mind where these properties would be essential?

8. Although the authors tried to explain some aspects of the emission of the films, they do not present a discussion on the importance behind the luminescence properties of this material.

9. As the material present some degree of persistent luminescence, I would like to know more about this specific mechanism in the referred system.

Response to Reviewer #1

Overview: *This manuscript describes the design and preparation of mechanical-robust room temperature phosphorescence materials through covalent click reaction. In this paper, the PVA-LDH composite has tensile strength of 97.9 MPa, 2.17-fold higher than pure PVA. There are many polymers and polymer composites with tensile strength higher than 100 MPa and even higher. It may be helpful to provide reasons for choosing PVA. It is known that PVA matrix has been frequently used by Y. Zhao and coworkers to fabricate polymer-based room-temperature phosphorescence materials. Other research group also use PVA to serve as matrix. Recently, Z. Yang and coworkers reported the preparation of triphenylene-2-ylboronic acid-PVA phosphorescence materials with lifetime above 3.0 s (Adv. Mater. 2022, 34, 2108333).*

Response: Thank you very much for your evaluation and valuable suggestions on our work. We appreciate that you agree to publish our manuscript after revision. Your comments are greatly helpful, and we would like to revise our manuscript according to your comments. All the changes are highlighted in yellow.

In our work, we have employed PVA as matrix due to the following reasons. First, PVA has been widely applied as matrix in constructing organic-inorganic composite films due to its flexibility, easy-preparation and low-cost. Previous works by Zhao or Yang's groups have shown the importance and wide application of PVA in preparing phosphorescent materials. This is the exact reason to study and strengthen the mechanical properties of these PVA-based films. Second, PVA is rich in hydroxyl groups, providing abundant reaction sites for B–O click reaction and thus the covalently crosslinked room-temperature phosphorescent materials. The contents of the hydroxyl groups could be adjusted for PVA with different alcoholysis degrees, leading to the reasonably regulated micro-structures and the macro-properties for the covalently crosslinked composites. Therefore, PVA was selected to construct inorganic-organic RTP composites with the chromophore BPBA and inorganic LDHs, and the

tensile strength of the composite film was significantly promoted based on the established covalent cross-linkage.

2. In this paper, boronic acid has also been used as phosphorescence dopant, whereas the phosphorescence lifetime is moderate (1.45 s, when compared to other boronic acid system, such as Adv. Mater. 2022, 34, 2108333 and Angew. Chem. Int. Ed.) and the phosphorescence quantum yield (3.92%) is low.

Response: These referenced papers have reported the RTP performances by employing a relatively high dopant concentrations of boronic acid, such as 2 wt% of boronic acid-related chromophores in *Adv. Mater.* **2022**, 34, 2108333 and 1 wt% in *Nat. Commun.* **2022**, 13, 347. In comparison, the doping contents of 4,4'-biphenyldiboronic acid (BPBA) in our work is only 0.1 wt%, which is 10- to 20-folds lower than the previous reports. Decent phosphorescence lifetime and quantum yield could be achieved for the composites at such a low dosage of boronic acid-related chromophores. Noteworthy that the phosphorescence performances of the composites in the presence of LDHs were promoted for ~5 folds higher than those in the absence of LDHs, ascribing to the inhibited non-radiation transition induced by the rigid LDHs. More importantly, BPBA with boronic acid acted as a bridge to connect organic PVA and inorganic LDHs through the B–O reaction, facilitating the simultaneously boosted mechanical robustness and RTP performance for the composite material. Therefore, we have proposed a covalent cross-linking strategy to engineer mechanical-robust RTP materials through the B–O click reaction between chromophores, PVA and LDHs, and it is believed that this strategy could be further utilized in other organic-inorganic system.

3. It is understandable that BPBA molecules with two boronic acid groups may serve as crosslinker for material fabrication. The statement of "covalent click reaction" need further evidence, for example, whether the reaction between boronic acid and other components exhibits the features of click chemistry in model system or this material system.

Response: Yes, the ionization constant of H^+ was usually employed to verify the success of

the B–O reactions (*J. Am. Chem. Soc.* 2018, **140**, 8611). In this work, the pH of BPBA was reduced from 10.86 to 10.54 after the reaction with LDHs and PVA, as a result of the transformation of the boronic acid to the boronic ester by B–O click reaction.

It has been reported that the B–O click reaction features the mild and rapid reaction process, and our preparation process was implemented under room temperature accordingly. Moreover, selectivity is another characteristic for the click reaction. We have employed two kinds of polymers in the absence of hydroxyl groups for the validation, such as positively charged poly(diallyldimethyl-ammonium chloride) (PDDA) and negatively charged poly(styrene-4-sulfonate) (PSS). It could be observed that the phosphorescence intensities of the composites prepared by PDDA or PSS are only 33% and 22% of that prepared by PVA (Supplementary Fig. 15), ascribing to the absence of the B–O reaction for PDDA and PSS. This fact validated the selectivity between boronic acid of BPBA molecules and hydroxyl groups of polymers. Taking advantages of the reaction selectivity, we have carried out the controlled experiments for PVA with different alcoholysis degrees and LDHs modified by SDS. When the hydroxyl groups were replaced or occupied in PVA or LDHs, the B–O reaction was inadequate and the phosphorescence performances were decayed. These results validated the features of B–O reaction between the boronic acid and hydroxyl groups in LDHs and PVA.

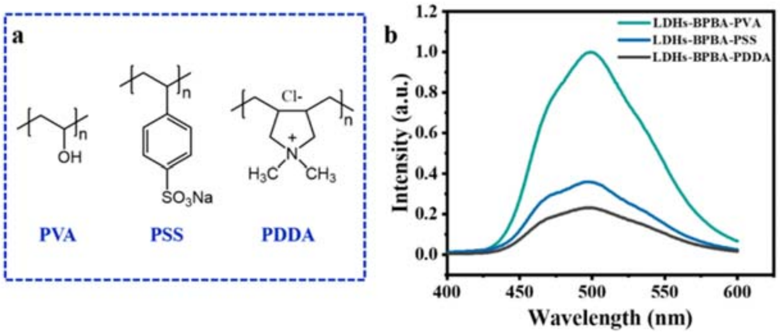

Supplementary Figure 15. a Molecular structure of PVA, poly(styrene-4-sulfonate) (PSS) and poly(diallyldimethyl-ammonium chloride) (PDDA), and (**b**) phosphorescence spectra of composites prepared by different polymers.

4. There are molecules with two or more boronic acid groups from commercial source or by chemical synthesis, among which there may be efficient and long-lived RTP dopants. In the reported studies, there are other polymer-based RTP and afterglow materials, responsive RTP materials, and materials with other afterglow mechanisms; some representative studies or review articles can be cited.

Response: Thank you for your suggestions. We have cited some corresponding references (*Nat. Commun.* **2021,** 12, 2297; *Small* **2023,** 19, e2207403; *Acc. Mater. Res.* **2021**, 2, 6544) as reference 3–5 as suggested in the revised manuscript.

5. In many polymer-based RTP systems, the RTP properties can only be obtained when the materials are sufficiently irradiated by excitation sources, because of the present of oxygen under ambient conditions. For the composite materials in this paper, it may be better to provide such information about RTP activation in the main text.

Response: To take the photos for the composites, we have used a 280 nm UV LED module to excite the fluorescence and phosphorescence, and the afterglow was recorded after irradiation for 3 s. Differently, no activation was implemented for the composites during the spectral and lifetime measurements. We have supplemented the above information in the experimental section as suggested.

Overall, the design strategy of RTP materials in this paper is important, whereas the selection of matrix and dopant for high-performance RTP materials, and the demonstration of covalent click reaction need major revision.

Response: Yes, we have finished revising the manuscript in detail in the current revision, and all the revisions were highlighted in yellow in the revised manuscript. The current manuscript has been greatly improved based on your comments. Thank you for your positive reports and helpful suggestions.

Reviewer #2

Overview: *The authors in this manuscript reported the design and construction of room temperature phosphorescence (RTP) with decent mechanical properties, based on the click reaction between chromophores, inorganic particle and polymer. The as-prepared composites showed decent RTP performance with lifetime to 1.45 s and simultaneously promoted mechanical strength of 97.9 MPa. These promotions could be ascribed to the established cross-linkage and covalent-bond-accommodated interfacial interaction in the polymeric composites. The proposed construction strategy could be applied in scalable preparation of RTP materials. Therefore, I recommend this work to be published in Nature Communications after the following minor revisions.*

Response: Thank you for recommending the publication of our manuscript after revisions. Your comments are greatly helpful and thus we would like to revise our manuscript according to your comments. All the changes are highlighted in yellow in the revised manuscript.

1. The authors have shown the significant effect of layered double hydroxides (LDHs) in the construction of RTP-efficient and mechanical-robust composites. To better illustrate this idea, the basic information of LDHs, such as the structure, composition and morphology, should be supplemented. What is the structural formula of LDHs? How are the hydroxyl groups arranged in the LDHs? I do think such an introduction is beneficial to understand the design strategy and the decent performances of the composites.

Response: Thank you for your suggestions. The LDHs possessed the general formula as $[M^{II}_{1-x}M^{III}_{x}(OH)_2]^{z+}A^{n-}_{z/n}\bullet yH_2O$ (*Chem. Soc. Rev.* **2014**, 43, 7040), in which M^{II}/M^{III} stand for the divalent/trivalent metal ions and A^{n-} represents the anion (Supplementary Fig. 1). In our work, we have employed MgAl-LDHs with the ratio of Mg/Al as 1.77 acquired by the inductively coupled plasma spectrometer. The LDHs showed layered structure as observed in the Fig. 2a and hexagonal platelet with lateral size around 2 μm (Supplementary Fig. 2). There are abundant hydroxyl groups attached on the host layer of LDHs, providing sufficient active

sites for the reaction with chromophores with boronic acid groups. We have supplemented the introduction of LDHs and the corresponding characterizations in the revised manuscript as suggested.

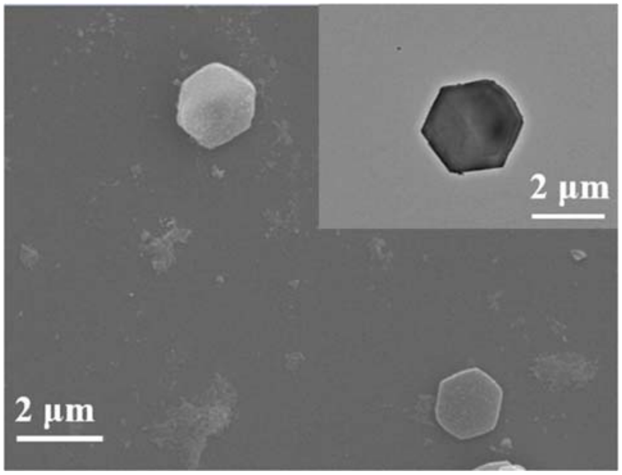

Supplementary Figure 2. SEM and TEM (inset) images of MgAl-LDHs.

2. To regulate the B-O covalent bonds, the authors have introduced sodium dodecyl sulfate (SDS) to modify the surface of LDHs. How could the SDS occupy the hydroxyl groups in LDHs? What is the interaction between SDS and LDHs?

Response: Yes, SDS is a kind of anionic surfactant with negatively charged sulfonate, and it can interact with the positively charged LDHs through electrostatic interaction. This demonstration could be evidenced by the zeta potential measurement. LDHs and SDS showed the averaged value of zeta potential as 40.93 mV and –38.83 mV from three parallel measurements, while reduced zeta potential of 8.46 mV could be acquired when the LDHs were modified by SDS (Supplementary Fig. 10). Accordingly, the SDS could attach on the surface of LDHs and occupy the active sites of hydroxyl groups, affecting the interaction between LDHs and chromophores with boronic acid groups.

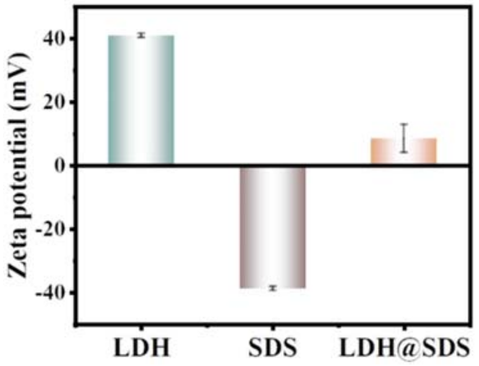

Supplementary Figure 10. Zeta potential measurements for LDHs, SDS and LDH@SDS.

3. The authors have introduced SDS to adjust the hydroxyl groups in LDHs and changed the alcoholysis degrees to regulate the hydroxyl groups in PVA in the "Regulation of the B–O covalent bonds" section. I recommend the authors to supplement the schematic representation for these regulations, so that the importance of the cross-linkage could be better understood.

Response: Thank you for your suggestion. The schematic representations for the regulation of the B–O covalent bonds based on the adjustment of LDHs and PVA have been supplemented in the revised manuscript as Supplementary Scheme 1. The schemes have shown that LDHs with modified SDS or PVA with low alcoholysis degrees exhibited decreased active sites to interact with boronic acid groups in of chromophores, leading to the decayed phosphorescence performances in Fig. 4.

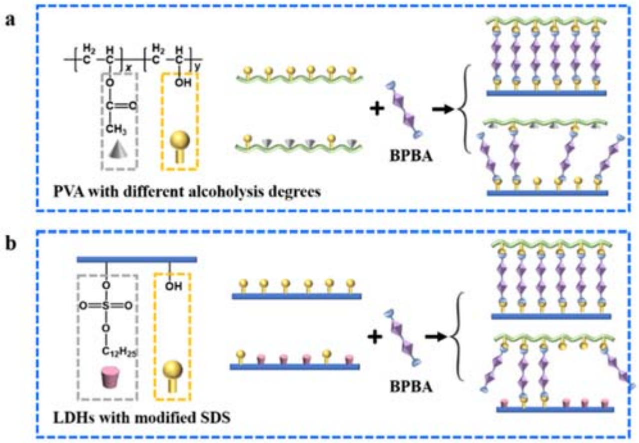

Supplementary Scheme 1. Schematic representation of the regulation of the B–O covalent bonds based on the adjustment of (**a**) PVA and (**b**) LDHs.

4. The fluorescence lifetime measurements for the composites should be provided in the supplementary material to support the data in the Table S1.

Response: Yes, we have supplemented the fluorescence lifetime decay curves for the composites as Supplementary Fig. 8. It could be observed that the fluorescence lifetime was promoted from 1.29 ns for BPBA molecules to the 2.80 ns for the 6%LDHs-BPBA-PVA film.

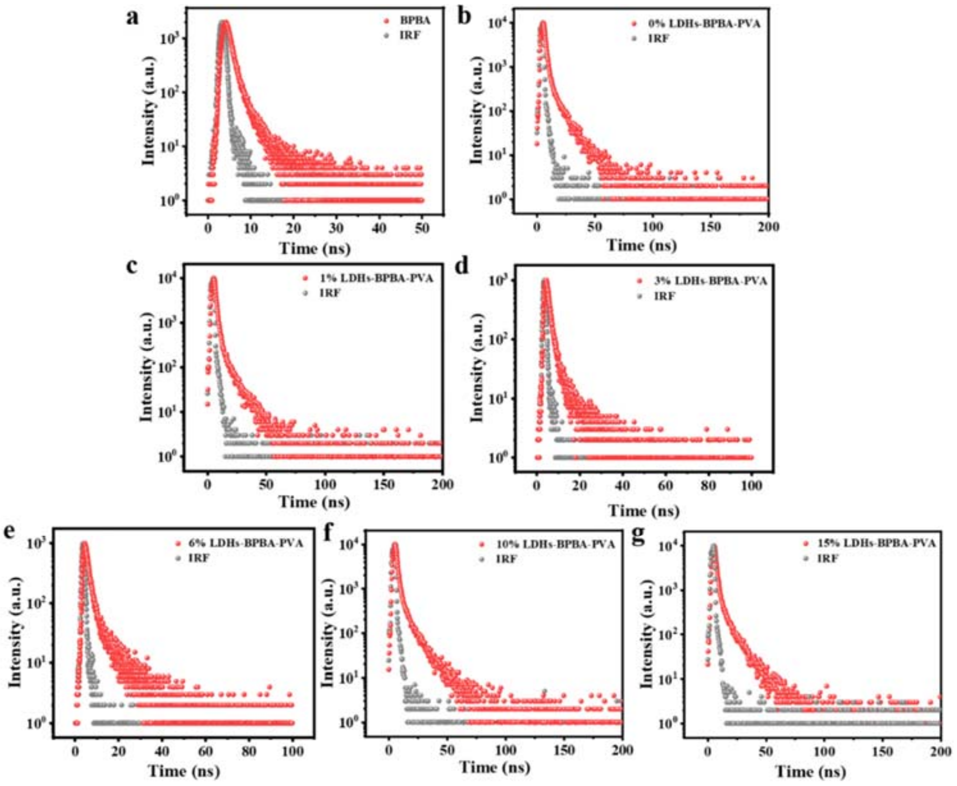

Supplementary Figure 8. Fluorescence lifetime measurements for (a) BPBA, (b) BPBA-PVA and *x*%LDHs-BPBA-PVA for (c) *x*= 1, (d) *x*=3, (e) *x*=6, (f) *x*=10, and (g) *x*=15.

5. How to measure the thickness of the films as shown in Fig. 6b?

Response: We have used a high-precision thickness gauge with an accuracy of 0.001 mm to measure the thickness of all the films, and the description has been supplemented in the revised manuscript.

6. As shown in Fig. 6a, the composite films also exhibited fluorescence responses towards mechanical deformations. How is the sensitivity of these fluorescence responses? Could the

authors explain the possible reason for the different response sensitivity between fluorescence and phosphorescence?

Response: Yes, we have supplemented the fluorescence responses of the 6%LDHs-BPBA-PVA film towards mechanical deformations, and the fluorescent emission spectra were recorded at the neck regions (position 1–4) and shoulder regions (position 5–8) of the films. The fluorescence intensities of the neck regions were higher than those of the shoulder regions (Supplementary Fig. 19). However, the fluorescence response is not as sensitive as the phosphorescence response as a result of the interferences of the background. In comparison, the composite films showed decent phosphorescence response towards mechanical deformations. Such a fact could be induced by the complex dissipation pathway of the triplet excitons which is more susceptible to respond towards the varied molecular arrangement of chromophores.

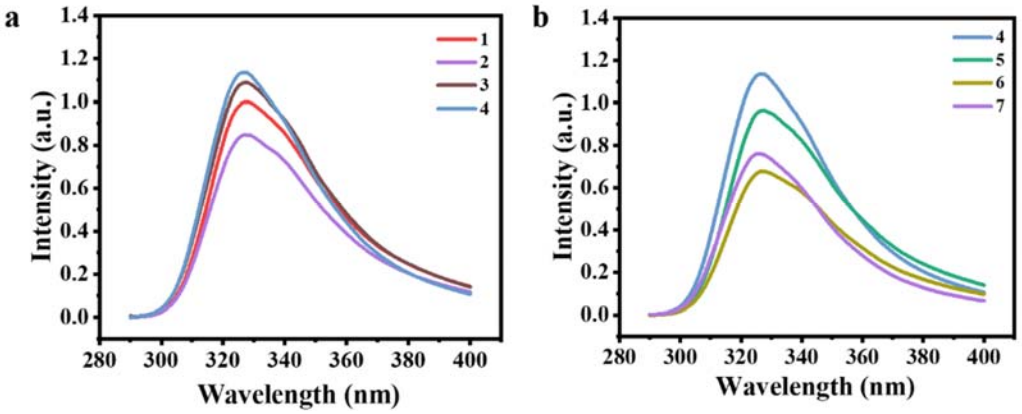

Supplementary Figure 19. Normalized fluorescent emission spectra of 6%LDHs-BPBA-PVA films stretched to the different lengths at the different positions: **(a)** positions 1–4 and **(b)** positions 4–7.

7. To arouse a broad interest from readers in this field, some related works on polymer hybrid RTP and wide applications (Chem. Sci. 2016, 7, 4519; Chem. Soc. Rev., 2021, 50, 5564; Chem. Eng. J. 2022, 430, 133084) could to be enriched as references.

Response: Thank you for your suggestions. We have cited these corresponding references as reference 6, 13 and 61 in the revised manuscript.

Reviewer #3

Overview: *The manuscript intended to be published at Nature Communication present a strategy to obtain a mechanical robust and flexible polymer composite based on the so-called B–O click reaction between inorganic and organic components.*

Before the manuscript becomes suitable for publication in such prestigious journal, I kindly request the authors to address the following and, in point of view, important questions:

Response: Thank you very much for your recommending the acceptance of our work after revisions. Your comments are greatly helpful and thus we would like to revise our manuscript according to your comments. All the changes are highlighted in yellow.

1. In the title, the authors use the term "Design of mechanical-robust room temperature phosphorescence materials". I my opinion, the title in its current form can mislead the readers towards understanding that the material is prepared at room temperature not the emission of the chromophores.

Response: Thank you for your comments. We have changed the title to "Design of mechanical-robust phosphorescence materials through covalent click reaction" according to your suggestion.

2. Concerning the LDHs, it is not clear for me which type was used. Was it obtained commercially? Who are the metals in the structure?

Response: Thank you for your suggestions. We have employed MgAl-LDHs in the work, with Mg and Al as M^{II}/M^{III} metal ions in the general formula of $[M^{II}_{1-x}M^{III}_{x}(OH)_2]^{z+}A^{n-}_{z/n}\bullet yH_2O$ for LDHs (*Chem. Soc. Rev.* **2017**, 46, 5950). The ratio of Mg to Al was determined as 1.77 by the inductively coupled plasma spectrometer. The preparation of MgAl-LDHs was implemented according to an established hydrothermal method. Specifically, $Mg(NO_3)_2\bullet 6H_2O$ and $Al(NO_3)_3\bullet 9H_2O$ provided the Mg and Al species in the host layer of LDHs, and urea was employed to provide CO_3^{2-} as the interlayer anionic of LDHs. These discussions have been supplemented in the revised manuscript.

3. The authors present the XRD pattern of the LDH showing some characteristic peaks. Indeed the 11.9° position correspond to the (003) crystal plane. What is the basal spacing associated to that? Who is the anionic molecule intercalated in this LDH?

Response: Yes, the 11.9° position correspond to the (003) crystal plane of LDHs. According to the Bragg equation, the associated basal spacing was calculated as d = 0.74 nm (*J. Phys. Chem. Solids*, **2006**, 67, 998), suggesting that the intercalated anionic molecule of this LDHs was CO_3^{2-}.

4. I found strange using the fact of the appearance of the 11.9° peak in the XRD pattern sufficient for the authors to state that the introduction of LDHs into the composite films was successful obtained. Is there another way to show that?

Response: Thank you for your suggestions. We have implemented the SEM and energy dispersive spectrometer (EDS) analysis for the 6%LDHs-BPBA-PVA films. The presence of Mg and Al elements from LDHs could be observed in the EDS analysis (Supplementary Fig. 5a). These characteristic Mg and Al elements distributed uniformly throughout the composite films as observed from mapping images in top-view (Supplementary Fig. 5b). Mapping analysis from side-view images also demonstrated the distribution of these elements along the z direction of the films (Supplementary Fig. 5c). Therefore, we can conclude the successful introduction of LDHs into the composite films.

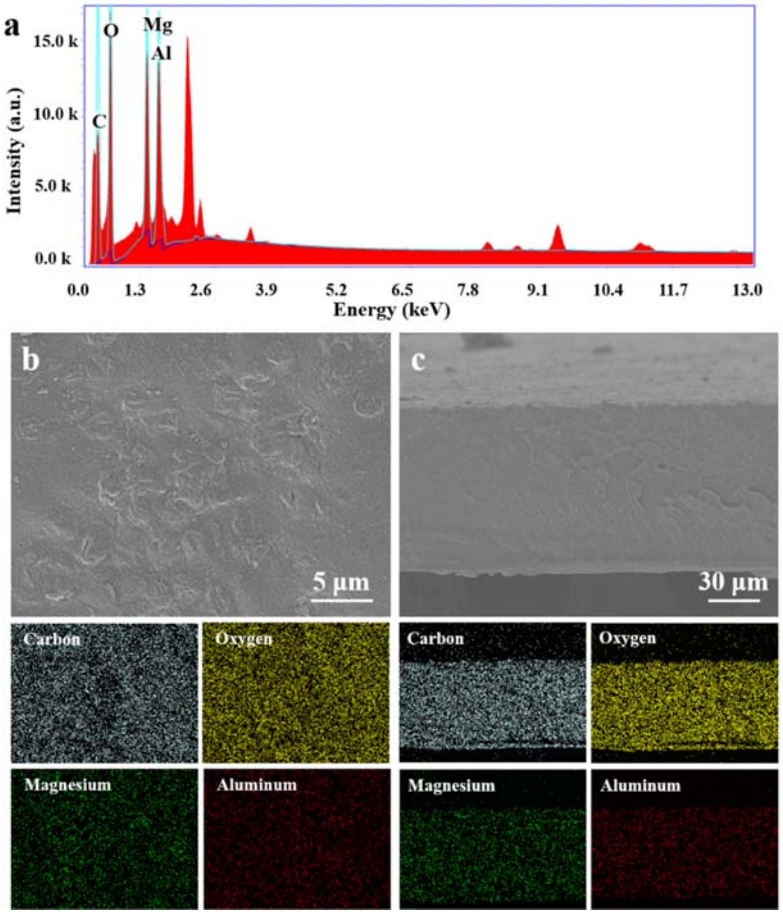

Supplementary Figure 5. a Element analysis, EDS mapping of oxygen, oxygen, magnesium and aluminum elements for 6%LDHs-BPBA-PVA films from (**b**) top-view and (**c**) side-view images.

5. Would be possible to apply a complementary experiment to confirm the so-called B-O click, meaning it there a way to study the coordination/environment of the B?

Response: Yes, the ionization constant of H^+ was usually employed to verify the success of the B–O reactions (*J. Am. Chem. Soc.* 2018, **140**, 8611). In this work, the pH of BPBA was reduced from 10.86 to 10.54 after the reaction with LDHs and PVA, as a result of the transformation of the boronic acid to the boronic ester by B–O click reaction. The occurrence of B–O reactions has also been validated by the IR spectra in the range of 1310 – 1430 cm^{-1}.

Moreover, XPS measurements for BPBA molecules and 6%LDH-BPBA-PVA composite film were implemented to study the variations of the coordination environment of B after the covalent reaction. It could be observed from the B 1s spectra that the binding energy shifted

from 191.80 to 191.94 eV after the BPBA were localized in the composite films (Supplementary Fig. 4a and 4b). The B 1s spectra could be further deconvoluted into the B−$(OH)_2$ (192.00 eV) and B−C (191.70 eV) bonds in the BPBA molecules, while B−O−C (192.40 eV), B−C (191.50 eV) and B−O−M(Al) (189.50 eV) could be observed in the composites. These variations suggested the successful interaction between BPBA, LDHs and PVA. Moreover, the new peaks of O−C (533.20 eV) and O−Al (531.30 eV) in O 1s spectra, and C−O (285.70 eV) in C 1s spectra, could be observed when the BPBA were incorporated in the composite films (Supplementary Fig. 4c−f). These results suggested the variations of the coordination environment of B and success of the B−O reaction in the composite films.

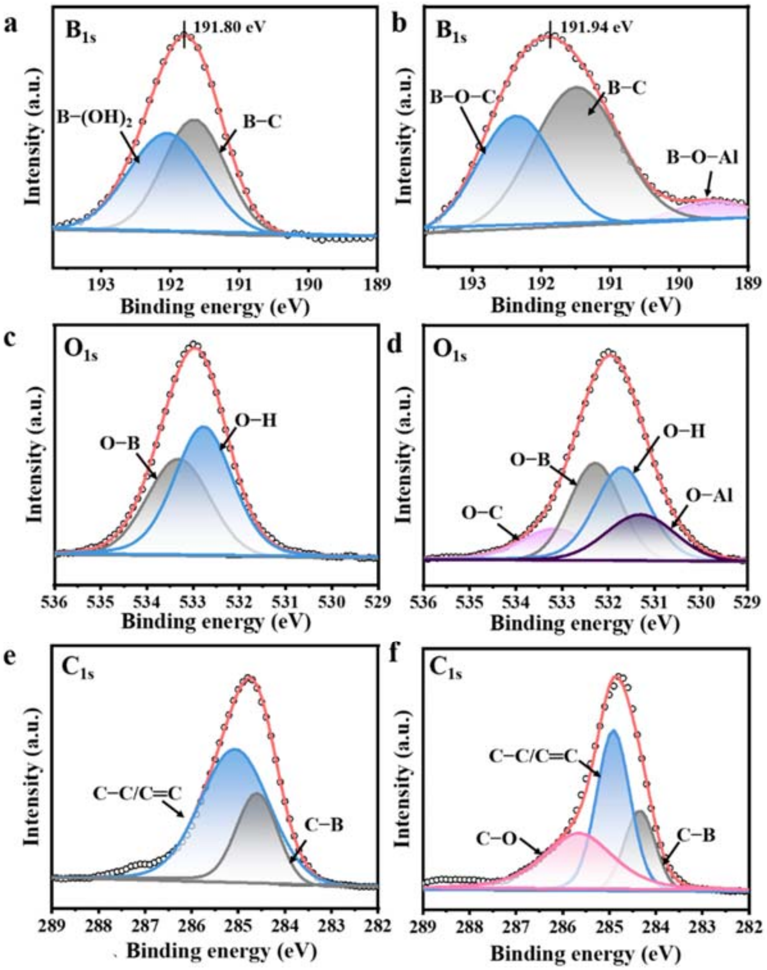

Supplementary Figure 4. XPS spectra of (**a** and **b**) B 1s, (**c** and **d**) O 1s, and (**e** and **f**) C 1s for (**a**–**c**) BPBA and (**d**–**f**) 6% LDHs-BPBA-PVA film.

6. Considering that LDH is a layered material, it is not clear for me where exact in the 4,4'-biphenyldiboronic acid (BPBA) molecules in that structure. Is it only in the external surface of the LDH? Choosing a different LDH with altered intercalated molecule would interfere in the process? Is the stacking number of Layers could also play a role?

Response: Yes, the BPBA molecules were attached on the external surface of LDHs. This is because that the XRD patterns of the composite films showed featured peaks of 2θ at 11.9°, in accordance with the pristine LDHs intercalated with CO_3^{2-} (Fig. 2a and Supplementary Fig. 3a). If the BPBA molecules were intercalated in the interlayer of LDHs, these 2θ peaks would shift to the lower degrees due to the larger size of BPBA molecules (about 15 Å from *Phys. Chem. Chem. Phys.* **2014**, 16, 8646) in comparison with the pristine CO_3^{2-}. No shift could be observed in the composites, suggesting that the BPBA molecules were only attached on the external surface of LDHs. Therefore, the BPBA molecules acted as a bridge between the inorganic LDHs and organic PVA to form a covalently cross-linked room-temperature phosphorescent materials as designed.

To figure out the influence of LDHs, we have synthesized LDHs intercalated with NO_3^- and prepared the LDHs-BPBA-PVA composites accordingly. It can be observed that the phosphorescence intensities of the composite prepared by LDH with NO_3^- or CO_3^{2-} are similar (Supplementary Fig. 13), suggesting that the anions in the interlayer of LDHs showed negligible effect on the phosphorescence performance for the composites. Similar results could be observed for the LDHs with the size of 50 nm prepared by colloidal milling (Supplementary Fig. 13 and 14). Moreover, we have employed an ultrathin LDH nanosheet for the preparation of composites, and the phosphorescence intensity was slightly decayed. Such a phenomenon could be ascribed to the existence of formamide which has been used to maintain the ultrathin morphology of the LDH nanosheets. Therefore, it could be concluded that the intercalated anions or the stacked layers of LDHs would not induce significant influence on the LDHs-BPBA-PVA composites, ascribing to the position of BPBA on the

surface of LDHs. In contrast, the modification of SDS on the surface of LDHs has induced critical effect on the performances of the LDHs-BPBA-PVA composites.

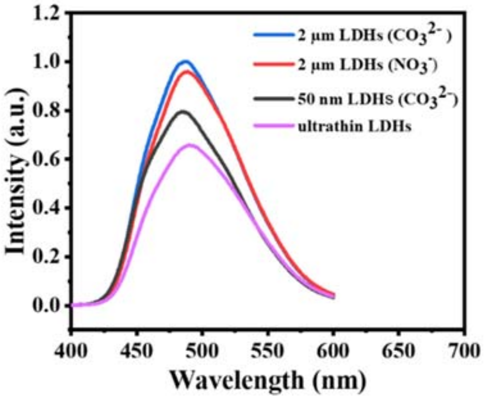

Supplementary Figure 13. Normalized phosphorescent emission spectra of LDHs-BPBA-PVA composites prepared by LDHs with different sizes and intercalated anions.

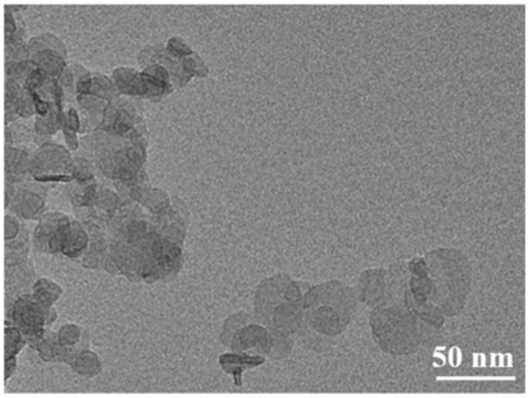

Supplementary Figure 14. TEM image of LDHs prepared by colloidal milling (50 nm CO_3^{2-}).

7. The authors state "luminescent polymeric materials should have good flexibility to meet the requirements for the applications". Which kind applications the authors have in mind where these properties would be essential?

Response: It has been reported that polymer-based phosphorescent materials with good flexibility, stretchability and easy processing could be widely used in many fields. The flexible phosphorescent films can be bent into various shapes and used as anti-counterfeiting devices and security labels (*Adv. Funct. Mater.* **2022**, 32, 2111941; *ACS Appl. Mater. Interfaces* **2022**, 14, 48133). For example, Wei Huang and coworkers have folded and bent

their flexible films to achieve a transparent three-dimensional object with multi-colored RTP, and they have demonstrated its potential application in anti-counterfeiting (*Nat. Commun.* **2023**, 14, 627). Yang Liu *et al.* have folded their flexible phosphorescent films into a variety of forms as windmills and bracelets with colorful afterglows. The flexibility of these materials enabled the development of a multi-level encryption platform that could be widely applied in different scenarios (*Mater. Horiz.* **2023**, 10, 197).

Moreover, the flexible luminescent polymeric materials could also be utilized as organic light-emitting diodes. These materials should be easily bent and folded, exhibiting elasticity resistant towards mechanical deformation and strain (*Adv. Funct. Mater.* **2018**, 28, 1802657; *Adv. Mater.* **2011**, 23, 5563; *Nat. Commun.* **2020**, 11, 6250). Therefore, it is anticipated that the luminescent polymeric materials should have good flexibility so that they could be widely used as the optical devices.

8. Although the authors tried to explain some aspects of the emission of the films, they do not present a discussion on the importance behind the luminescence properties of this material.

Response: Thank you for your suggestion. The luminescence properties of the composite films could be utilized to understand the arrangement of the molecules and the structures of the covalently crosslinked network. In this work, the BPBA with boronic acid groups acted not only as chromophores in the composites, but also as a bridge to connect the inorganic and organic phases through the covalent bonding structures. When the boronic acid groups attached on the two sides of BPBA molecules reacted with LDHs and PVA, the BPBA molecules were immobilized in a covalently crosslinked network. Accordingly, the molecular motions of BPBA were effectively inhibited, leading to the decayed non-radiative transition and thus the boosted luminescence behaviors. These promoted luminescence properties of the composites demonstrated the success of the B–O interaction that responsible for covalently cross-linked network and localized arrangement of BPBA molecules. In addition, the loading stress of the composites could be dissipated through the bridged BPBA to the rigid inorganic

sheets, contributing to the decent mechanical properties of the composites. These facts addressed the ability of the luminescence properties to understand the microstructures and promotion mechanism in the covalently cross-linked composites. Therefore, we have provided an inorganic-organic luminescence composite that featured the advantages of easy processability, flexibility, mechanical robustness, and scalability. It is anticipated that these prepared composites could be wide applied as data encryptors, electronic displays and flexible sensors. We have supplemented the corresponding descriptions in the revised manuscript as suggested.

9. As the material present some degree of persistent luminescence, I would like to know more about this specific mechanism in the referred system.

Response: First, pure BPBA molecules are thought to be the origin of phosphorescence. Except for the π-π* transition presented in the biphenyl moiety, the BPBA molecules exhibited characteristic n-π* transition that O atoms in the boric acid groups provided n electrons conjugated with the phenyl moiety (*Angew. Chem. Int. Ed.* **2021**, 133, 9586). The pristine BPBA molecules could emit weak phosphorescence at room temperature. When the BPBA molecules were covalently crosslinked with LDHs and PVA, the n-π* transition of boric acid group to phenyl group was facilitated. This condition has been confirmed by the largely promoted absorbance at 350 nm for LDHs-BPBA-PVA composites, in comparison with the pristine BPBA and BPBA-PVA (Supplementary Fig. 9). In other words, the B–O covalent crosslinking network facilitated the intersystem crossing from singlet to triplet states, resulting in the promoted phosphorescence behaviors in the LDHs-BPBA-PVA composites.

Second, the inhibition of the molecular relaxation is critical to construct ultra-long room-temperature phosphorescent materials, due to the easily dissipated energy as non-radiative transitions of triplet excitons. To inhibit the dissipation of triplet excitons for the BPBA molecules, a rigid network based on the B–O covalent bonds was established between BPBA, inorganic LDHs and PVA matrices This B–O covalent network has been confirmed by

XRD pattern (Fig. 2a), FT-IR (Fig. 2b) and XPS spectra (Supplementary Fig. 4) as discussed in the manuscript. Accordingly, the rate constants for the non-radiative transition of phosphorescence was reduced from 1.07 s^{-1} for BPBA-PVA to 0.64 s^{-1} for 6%LDHs-BPBA-PVA.

In addition, it has been reported that triplet excitons could be easily quenched by oxygen in the environment. LDHs showed excellent oxygen barrier performances in the composites, preventing the diffusion of ambient oxygen in the membrane and suppressing the quenching of triplet excitons (*Adv. Funct. Mater.* **2014**, 24, 514). Based on these mechanisms, persistent luminescence could be achieved for the LDHs-BPBA-PVA composites. We have supplemented the corresponding descriptions in the revised manuscript as suggested.

Reviewers' Comments:

Reviewer #1:
Remarks to the Author:
The current form of this manuscript is better than the previous version.

Reviewer #2:
Remarks to the Author:
The authors have done essential revision with necessary experiments. Their responses appear satisfactory. Therefore, I recommend acceptance of this article.

Reviewer #3:
Remarks to the Author:
In the revised manuscript "Design of mechanical-robust room temperature phosphorescence materials" the authors were able to respond most of the important questions initially addressed. For this reason, I considered now the manuscript suitable for publication in Nature Communication.

Response to Reviewer #1

Overview: *The current form of this manuscript is better than the previous version.*

Response: Thank you very much for your positive appraisal to our work.

Response to Reviewer #2

Overview: *The authors have done essential revision with necessary experiments. Their responses appear satisfactory. Therefore, I recommend acceptance of this article.*

Response: Thank you for your recommending acceptance of the current version of our manuscript.

Response to Reviewer #3

Overview: *In the revised manuscript "Design of mechanical-robust room temperature phosphorescence materials" the authors were able to respond most of the important questions initially addressed. For this reason, I considered now the manuscript suitable for publication in Nature Communication.*

Response: We appreciate that you recommend publishing our manuscript in the current version.
